# End-to-End AUV Motion Planning Method Based on Soft Actor-Critic

**DOI:** 10.3390/s21175893

**Published:** 2021-09-01

**Authors:** Xin Yu, Yushan Sun, Xiangbin Wang, Guocheng Zhang

**Affiliations:** Science and Technology on Underwater Vehicle Laboratory, Harbin Engineering University, Harbin 150001, China; 505779480@hrbeu.edu.cn (X.Y.); sunyushan@hrbeu.edu.cn (Y.S.); zhangguocheng@hrbeu.edu.cn (G.Z.)

**Keywords:** autonomous underwater vehicle (AUV), deep reinforcement learning (DRL), soft actor–critic (SAC), generative adversarial imitation learning (GAIL), motion planning

## Abstract

This study aims to solve the problems of poor exploration ability, single strategy, and high training cost in autonomous underwater vehicle (AUV) motion planning tasks and to overcome certain difficulties, such as multiple constraints and a sparse reward environment. In this research, an end-to-end motion planning system based on deep reinforcement learning is proposed to solve the motion planning problem of an underactuated AUV. The system directly maps the state information of the AUV and the environment into the control instructions of the AUV. The system is based on the soft actor–critic (SAC) algorithm, which enhances the exploration ability and robustness to the AUV environment. We also use the method of generative adversarial imitation learning (GAIL) to assist its training to overcome the problem that learning a policy for the first time is difficult and time-consuming in reinforcement learning. A comprehensive external reward function is then designed to help the AUV smoothly reach the target point, and the distance and time are optimized as much as possible. Finally, the end-to-end motion planning algorithm proposed in this research is tested and compared on the basis of the Unity simulation platform. Results show that the algorithm has an optimal decision-making ability during navigation, a shorter route, less time consumption, and a smoother trajectory. Moreover, GAIL can speed up the AUV training speed and minimize the training time without affecting the planning effect of the SAC algorithm.

## 1. Introduction

Autonomous underwater vehicles (AUVs) with their autonomy and flexibility play an important role in seabed surveying and mapping, ocean monitoring, underwater structure survey, information collection, and other aspects with the continuous advancement of computer software and hardware and the artificial intelligence (AI) technology in modern times. Intelligence is the overall development trend of the AUV technology, and motion planning technology is the basis for AUV to autonomously navigate and complete various tasks.

### 1.1. Background

Motion planning is guided by global path planning, using local environment information obtained online by sensing devices and generating discrete or continuous spatial path points or control information at the bottom of the robot, allowing the planning of the position, speed, and acceleration of the AUV during its motion. This task needs to satisfy two conditions. (1) Completeness: when all types of constraints are satisfied, the system can successfully plan a collision-free route to the target point. (2) Optimality: the shortest route must be taken, and the least time or energy consumption must be expended while completing the basic planning tasks.

In the actual process, the AUV motion planning task has the following difficulties:

- AUV motion planning becomes a difficult problem in navigation due to the uncertainty of the marine environment, the system dynamic constraints of the AUV itself, and the limitations of obstacle avoidance sonar and other sensor devices in the perception of the marine environment.
- In many early methods, the motion planning task is divided into two parts, namely, path planning and following, and the design process is complicated. These two modules depend on the characteristics of the environment and the dynamic constraints of the system, resulting in a sensitive system. Therefore, the robot can only obtain strategies in a single environment and lacks adaptability to the environment.
- Most existing methods have low exploration ability, easily fall into the local optimum, and cannot achieve the goal of the AUV motion planning task under the condition of multiple constraints.

RL is a research hotspot in the field of machine learning. In recent years, RL has been successfully applied to the fields of robot planning, control, and navigation. This field aims to make the agent continuously learn in the process of interaction with the environment and judge the rationality of action selection by calculating the cumulative expected return value after executing the action in the current state. End-to-end learning can be achieved by combining with deep learning (DL). Specifically, the input-to-output mapping relationship can be directly constructed through the model, which can save a substantial amount of time. The application of deep reinforcement learning (DRL) [1] technology in AUV motion planning research can give full play to the advantages of reinforcement learning. A series of decision sequences that consider long-term effects can be generated through self-interactive training, which can greatly improve the robustness and adaptability of the AUV to complex environments.

However, the application of DRL in AUV motion planning tasks is still in early development, and the following difficulties have been encountered:

- DRL is an unsupervised method. Some algorithms require tens of millions of interactions with the environment to learn a successful strategy. The training speed is considerably slow, and the cost is high. This problem is also aggravated by the large number of hyperparameters that need to be adjusted.
- The reward function is difficult to set, and the quality of the design directly affects the training success. However, the reward function at this stage is only independently formulated by the researcher according to the research problem, and no guiding rule has been established. Furthermore, AUV motion planning is a sparse reward task. In the initial stage of the training process, the robot cannot easily obtain a positive reward value, resulting in a complicated training.

### 1.2. Related Work

The following are the categories of AUV motion planning algorithms in the process of continual development. (1) Methods based on geometric model search (visualization, Dijkstra, A*, D* algorithm, etc.) [2,3]: we need to build a geometric model of the planning environment to use this type of method. The trajectory planned by this method is not smooth and cannot meet the maneuverability of the AUV. Every new plan must be calculated from scratch, and there is a lack of intelligent understanding of motion planning. (2) Sampling-based method (rapidly exploring random tree) [4]: the method can search high-dimensional space quickly and effectively, but it also has disadvantages, such as the planning result not being optimal and the planned path not being smooth enough. (3) Artificial potential field method [5]: this method requires less environmental information and is convenient to operate; however, it easily falls into the local optimum problem. (4) Curve interpolation method (Bezier curve, polynomial curve, B-spline curve, Dubins curve, etc.) [6,7]: this type of algorithm is relatively intuitive, the planned trajectory is also very smooth, and the curvature changes continuously. The disadvantage is that the calculation is large, the real-time performance is not good, and it is difficult to find the best evaluation function. (5) Method based on AI: this mainly includes ant colony algorithm, genetic algorithm, and reinforcement learning methods (RL) [8,9,10]. The main advantages of these algorithms are that we do not need to build a complicated environmental obstacle model. AUV can perform random or specific search in the space of the environment and deal with various complex problems under multiple constraints.

At present, a large amount of literature has studied motion planning based on AI. Bai et al. [11,12] proposed a co-evolutionary multi-population genetic algorithm and several clustering-based algorithms, which solves the problem of time optimality in multi-vehicle task assignment in the drift field by combining with optimal control theory. Li et al. [13] proposed an improved ant colony algorithm. The improved method sets the initial pheromone concentration based on the location information of the current feasible target point and the end point, which solves the problem of AUV path planning, accelerates the convergence speed of training, and reduces the generation of local optimal solutions. Camci [14] proposed an end-to-end motion planning system based on DRL for quadrotor. This system uses the original depth image obtained from the front camera and directly generates a local motion plan in the form of smooth motion primitives while avoiding obstacles and moving to the target. However, the camera cannot easily capture a clear image in the underwater complex environment. The image is difficult to map to the control policy of the AUV. Doukhi [15] designed an end-to-end local motion planning system for autonomous aerial robots based on RL, which directly maps the noisy robot’s own state and laser scanning measurement to continuous motion control to complete the navigation task to reach the target point. The obtained policy can perform collision-free flight in the real world. Cheng et al. [16] used convolutional neural networks to extract the characteristics of sensor information and designed a comprehensive reward function related to obstacle avoidance, target approach, speed correction, and attitude correction. The obstacle avoidance problem of underactuated unmanned marine vessels was realized on the basis of the deep Q-network (DQN) architecture. Then Sun et al. [17] proposed an AUV path planning method in a three-dimensional environment based on the DQN framework and combined with the prioritized experience replay. Sun et al. [18] realized multi-constraint motion planning of AUV in an unknown environment based on the proximal policy optimization (PPO) algorithm. This mechanism can realize a single-step update and can face discrete and continuous controls compared with the traditional policy gradient method. However, PPO is an on-policy algorithm that requires considerable sampling to learn, resulting in low sample efficiency. Butyrev et al. [19] proposed a mobile robot motion planning method based on the Deep Deterministic Policy Gradient (DDPG) algorithm. The system takes the position, speed, and direction of the robot as inputs and considers the kinematic and dynamic constraints of the robot. Moreover, the system randomly generates the target state and outputs the actual motion speed and steering angle, thereby solving the problem of motion planning in the continuous state and motion space. However, DDPG is a deterministic policy algorithm because only the optimal action is considered in each state, and the robot will only find an optimal path.

Haarnoja [20,21] released the soft actor–critic (SAC), which is suitable for the real-world robot skill learning and can be highly consistent with the requirements of robot experiments. In recent years, SAC has been widely used in autonomous decision-making, intelligent planning, and motion control of mobile robots, UAVs, and manipulators. Prianto et al. [22] presented a deep reinforcement learning-based path planning algorithm for the multi-arm manipulator. To solve the problem of high-dimensional path planning, SAC is used to enhance the exploration performance of the robotic arm. The experiments show that the algorithm can find the shortest path at any starting and target positions, and the generated path is shorter and smoother than the existing results. Wong et al. [23] designed a SAC-based motion planning method for a dual-arm robot with two seven-degrees-of-freedom manipulator arms, which enables the robot to effectively prevent self-collision while avoiding the joint limits and singularities of the manipulator arm. Liu et al. [24] proposed a mobile robot planning and navigation method through deep reinforcement learning in a dense pedestrian environment. First, the A* algorithm is used for global path planning. Subsequently, the SAC algorithm is used to make action decisions along the nodes generated on the global path. Cheng [25] successfully solved the autonomous decision-making problem of drones based on the SAC algorithm. The experimental results show that the drone can learn to change the trajectory in a short time through the SAC algorithm to avoid being intercepted and adjust the trajectory to successfully land to the target point. In the field of autonomous driving of automobiles, the SAC-based deep-enhanced planning system can handle various complex scenarios [26,27]. However, SAC is rarely used in the field of underwater vehicles at present.

The pros and cons of various algorithms for AUV motion planning are shown in Table 1. SAC has the following advantages compared with other algorithms: (1) SAC is an off-policy algorithm; (2) the performance of this algorithm is less sensitive to different hyperparameter values than other RL algorithms, thereby greatly reducing the time spent in adjusting hyperparameters; (3) SAC is a DRL algorithm based on maximum entropy, which can greatly enhance the exploration ability of AUV. In a complex and changeable environment, such as underwater, SAC can also find the optimal solution of the planning and complete the specified task under multiple constraints [28]. 

We often encounter a problem in the process of using reinforcement learning to train agents. Learning a policy from scratch is difficult and time-consuming [29]. A good solution is imitation learning (IL). This method enables the agent to learn the expert’s policy from the expert’s demonstration. The following are two main ways to solve the imitation problem: the first way is behavioral cloning (BC) [30], which takes the policy as a supervised learning problem to learn state-action pairs from expert trajectories. The BC method has been used to successfully learn many different missions, such as quadrotor navigation [31] and autonomous ground vehicle navigation [32]. The operation of BC is simple; however, it will only succeed in the case of a large amount of data [33,34] due to the compound error caused by the covariate shift [35]; hence, there are certain limitations. The second method is inverse reinforcement learning (IRL) [36]. IRL essentially learns a cost function under which the expert policy is the only optimal one, which can avoid the covariate shift problem in BC. In recent years, IRL has been applied in a range of fields, from predicting the behavior of taxi drivers [37] to planning the steps of quadruped robots [38]. However, IRL are typically computationally expensive in the recovery of expert cost function. 

Generative adversarial networks (GAN) have been successful in image generation [39,40] and have inspired similar methods of imitating behavior. Generative adversarial imitation learning (GAIL) [41] introduces the GAN into IRL and imitates the behavior of experts through direct policy optimization rather than learning the cost function first [42]. GAIL consists of a generator that generates the distribution of state-action pairs and a discriminator that distinguishes the generated distribution from the target constructed by expert demonstration samples. The reward function of GAIL encourages the generator to confuse the discriminator and aims to learn close-to-optimal behaviors directly from expert demonstrations and self-exploration [43,44]. The discriminator network is trained to distinguish agent and expert behavior through observation and used as a reward function. GAIL allows agents to overcome exploration challenges through the use of expert demonstrations, while also making it possible to achieve high asymptotic performance. GAIL has been applied in the certain fields, such as autonomous driving, mobile robot map coverage, robot motion planning, and joint control, and has achieved good results [45,46].

### 1.3. Proposed Solution

The main contributions of this research are as follows. This research designs an end-to-end AUV motion planning method based on the SAC algorithm to solve the problems of poor exploration ability, single strategy, and high training cost in AUV motion planning task in the background and overcome certain difficulties, such as multiple constraints and a sparse reward environment. Furthermore, the GAIL method is used to assist its training, and the AUV is directed by experts, which reduces the cost of interaction between the AUV and the environment and solves the difficult and time-consuming problem of learning strategies from scratch in reinforcement learning. Therefore, the proposed SAC-GAIL algorithm not only exerts the high exploratory nature of SAC but also has the characteristics of rapid convergence of GAIL. Finally, a comprehensive reward function related to speed, distance, and heading angle is designed to avoid the problem of sparse reward.

In addition to the facts described above, the research process also has the following difficulties:

- Useful samples and expert demonstrations for AUV are difficult to collect in IL. The quality of the demo also has a greater impact on the training effect.
- GAIL introduces a survivor bias in the learning process, which encourages the agent to live as long as possible by giving positive rewards based on the similarity with the expert, which directly conflicts with goal-oriented tasks. In this case, the proportions of GAIL signals and external rewards are difficult to coordinate.

The rest of this paper is organized as follows. Section 2 constructs the maneuverability model of AUV and formulates the motion planning problem. This study directly maps the state information of the AUV and the environment into the control instructions of the AUV based on the DRL method, realizing the end-to-end processing of the information. Section 3 determines the state and action spaces of the AUV. GAIL is used to guide the AUV to imitate the behavior of experts based on the deep reinforcement learning framework of SAC, and an AUV motion planning system based on SAC-GAIL is proposed. Finally, the setting of reward function is introduced in detail. A simulation experimental platform is built to verify the end-to-end motion planning system proposed in this paper and analyze the experimental results in Section 4. Section 5 presents the conclusions.

## 2. AUV Model and System Description

### 2.1. Preliminaries

**Figure 1:**
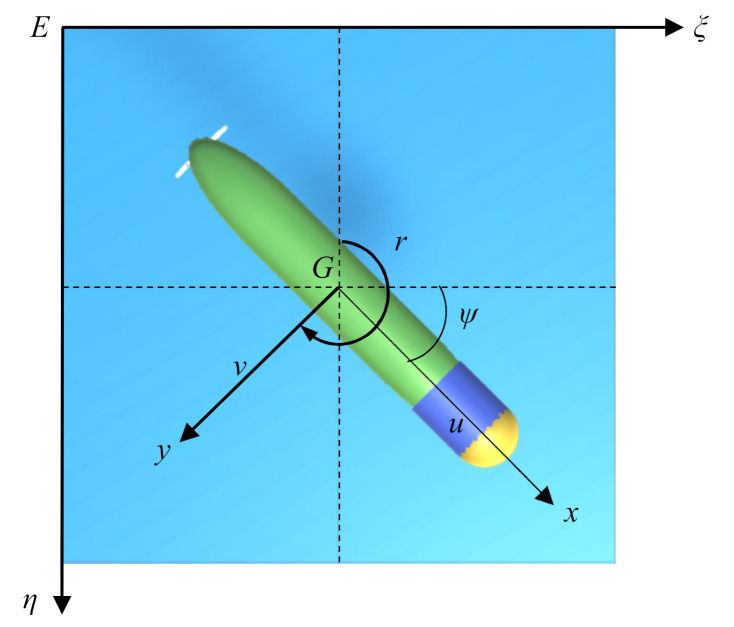
Major components of the AUV.

Experiments were performed using a small AUV (Figure 1). The length of the AUV was 1.46 m, and its mass was 45 kg. The AUV was mainly equipped with underwater detection sensors, such as a doppler velocity log (DVL) and an obstacle avoidance sonar (OAS), to detect the environment based on its own information. DVL can obtain the speed information of AUV, and OAS can acquire the position information of the obstacle and target point. The parameter information of AUV and sensor is shown in Table 2 and Table 3.

Only the horizontal motion of AUV is considered in this work. The horizontal motion of the AUV is composed of three parts: surge, sway, and yaw. The main definitions of motion symbols in this research adopt the symbols recommended by the International Towing Tank Conference, as shown in the figure. The state of the AUV can be represented by vector $\upsilon ={\left[u,v,r\right]}^{T}$ and $\eta =\left[x,y,\psi \right]$, which represent the speed information and position information of the AUV, respectively. ψ is the heading angle of the AUV, and $\left[x,y\right]$ is the position in the earth-fixed inertial frame. The linear velocities ${\left[u,v,r\right]}^{T}$ correspond to surge and sway, and yaw in the body-fixed frame of the AUV. Only the horizontal motion is considered in this research, which means that the vertical velocity, roll velocity, and pitch velocity of the AUV are ignored. In this case, the nonlinear motion equation of AUV can be described as follows [47]:

(1) $$\dot{\eta }=R\left(\psi \right)\upsilon ,$$

(2) $$M\dot{v}=\tau -C\left(v\right)v-D\left(v\right)v-g\left(\eta \right)+\tau_{w},$$

where $R\left(\psi \right)$ is the three degree of freedom coordinate transformation matrix of AUV horizontal motion, which has the following properties: $R{\left(\psi \right)}^{T}R\left(\psi \right)=I$, and for all ψ: $\left\|R\left(\psi \right)\right\|=1$. In general, $\frac{d}{dt}\{R\left(\psi \right)\}=\dot{\psi }R\left(\psi \right)S$, where

(3) $$R\left(\psi \right)=\left[\begin{array}{ccc} \cos\left(\psi \right) & -\sin\left(\psi \right) & 0\\ \sin\left(\psi \right) & \cos\left(\psi \right) & 0\\ 0 & 0 & 1 \end{array}\right],\;S\overset{3DOF}{=}\left[\begin{array}{ccc} 0 & -1 & 0\\ 1 & 0 & 0\\ 0 & 0 & 0 \end{array}\right].$$

The system inertia matrix $M=M_{A}+M_{RB}>0$ is the combination of added mass matrix $M_{A}$ and rigid-body matrix $M_{RB}$. The surge, sway, and yaw motions are decoupled because of the symmetry of the inertia matrix of the system. The added mass matrix of AUV can be obtained as follows:

(4) $$M_{A}=\left[\begin{array}{ccc} -X_{\dot{u}} & 0 & 0\\ 0 & -Y_{\dot{v}} & -Y_{\dot{r}}\\ 0 & -Y_{\dot{r}} & -N_{r} \end{array}\right].$$

AUV is commonly assumed to have a homogeneous mass distribution and an xz-plane symmetry; hence, $I_{xy}=I_{yz}=0$. Let the body-fixed frame coordinate origin be set in the centerline of the AUV in the point; accordingly, $y_{g}=0$. Let $x_{g}=0$ to simplify the process. Based on the above assumption, the matrix related to rigid body motion is simplified as follows:

(5) $$M_{RB}=\left[\begin{array}{ccc} m & 0 & 0\\ 0 & m & 0\\ 0 & 0 & I_{z} \end{array}\right].$$

After merging, we the following results are obtained:

(6) $$M=\left[\begin{array}{ccc} m-X_{\dot{u}} & 0 & 0\\ 0 & m-Y_{\dot{v}} & -Y_{\dot{r}}\\ 0 & -Y_{\dot{r}} & I_{z}-N_{\dot{r}} \end{array}\right],$$

where $I_{*}$ is the moment of inertia of the AUV.

$C\left(v\right)$ represents the Coriolis centripetal force matrix and has the following properties: $C\left(v\right)=-C{\left(v\right)}^{T}$. The anti-symmetric matrix is also composed of two parts:

(7) $$C\left(v\right)=C_{A}\left(v\right)+C_{RB}\left(v\right),\;$$

where:

(8) $$C_{A}\left(v\right)=\left[\begin{array}{ccc} 0 & 0 & Y_{\dot{v}}v+Y_{\dot{r}}r\\ 0 & 0 & -X_{\dot{u}}u\\ -Y_{\dot{v}}v-Y_{\dot{r}}r & X_{\dot{u}}u & 0 \end{array}\right],$$

(9) $$C_{RB}\left(v\right)=\left[\begin{array}{ccc} 0 & 0 & -mv\\ 0 & 0 & mu\\ mv & -mu & 0 \end{array}\right],$$

where $M=M^{T}$, $C_{RB}\left(v\right)=-C_{RB}{\left(v\right)}^{T}$, and $C_{A}\left(v\right)=-C_{A}{\left(v\right)}^{T}$. $D\left(v\right)$ is the hydrodynamic damping matrix of AUV, which can be expressed as follows:

(10) $$D\left(v\right)=-\left[\begin{array}{ccc} X_{u\left|u\right|}\left|u\right| & 0 & 0\\ 0 & Y_{v\left|v\right|}\left|v\right|+Y_{v\left|r\right|}\left|r\right| & Y_{r\left|v\right|}\left|v\right|+Y_{r\left|r\right|}\left|r\right|\\ 0 & N_{v\left|v\right|}\left|v\right|+N_{v\left|r\right|}\left|r\right| & N_{r\left|v\right|}\left|v\right|+N_{r\left|r\right|}\left|r\right| \end{array}\right],$$

where $g_{\eta }$ is the force and moment produced by gravity and buoyancy; however, it is ignored because only the plane motion of AUV is considered in this research.

τ denotes the vector of the control input. The AUV studied in this research is underactuated, and the number of system inputs is less than that of motion degrees of freedom. Specifically,

(11) $$\tau =\left[\begin{array}{ccc} \tau_{u} & 0 & \tau_{r} \end{array}\right],$$

where $\tau_{u}$ and $\tau_{r}$ represent the surge force and yaw moment, respectively.

### 2.2. Problem Formulation

The motion planning of AUV is a complex multi-constraint problem, and its basic task is to avoid obstacles while reaching the target point. In the actual movement process, the sensor is required to transmit information, $s_{t}\in S^{34}$, about the environment and its own state to the AUV and output the planning policy. According to the dynamic equation of the AUV, the propeller outputs surge force and yaw moment to control the motion of the underactuated AUV. Therefore, the end-to-end AUV motion planning system proposed in this research directly maps the state information $s_{t}$ to the AUV’s actions $a_{t}=\left(\tau_{u},\tau_{r}\right)\in A^{2}$ at each moment. Therefore,

(12) $$a_{t}=\left(\tau_{u},\tau_{r}\right)=f\left(s_{t}\right)\in A^{2}.$$

(13) $$s_{t}=\left(x_{t},v_{t},o_{t}\right).$$

The input information $s_{t}$ for AUV motion planning includes the position information $x_{t}$ of the target point and AUV, the actual velocity information $v_{t}$ of AUV, and the obstacle information $o_{t}$ detected through the obstacle avoidance sonar. In this research, sensor information fusion and filtering are not discussed.

**Figure 2:**
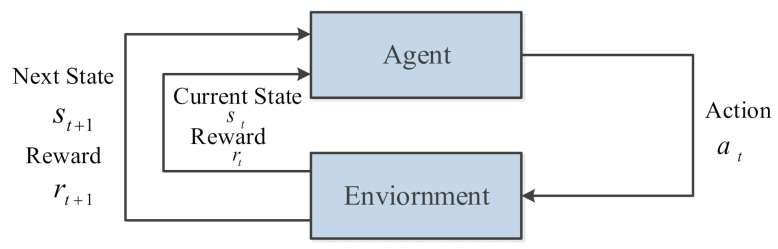
Markov decision process.

In Figure 2, the planning system based on the RL algorithm gives the AUV a certain reward value $r_{t}$ according to the quality of the behavior to adjust the probability of performing each action, and the AUV will be in the next state. DRL is employed to update the strategy $\pi_{\theta }$ by adjusting the weight of the neural network $w_{i}$. The above process is repeated, and the AUV will continue to interact with the environment until the optimal policy $\pi_{\theta }^{*}$ is obtained, which is a complete Markov decision process (MDP) [48]. AUV can obtain a complete smooth trajectory $Trajectory=\left(s_{0},a_{0},s_{1},a_{1},\ldots \ldots ,s_{end}\right)$ through a series of state action sequences, where $s_{end}$ is the terminal state. 

## 3. Method

### 3.1. State Space and Action Space of AUV

The AUV acquires the observation vector according to the state space it is in, takes it as the input value of the neural network, and makes the corresponding decision through the motion planning system. The AUV needs to be provided with the right information for it to successfully learn the task. A good rule of thumb for deciding what information to gather is to consider all the data needed to arrive at a solution to the task. However, the size of the observation vector should also be limited to a certain range because the input of the neural network becomes larger with the increase in the AUV state space dimension. Moreover, the algorithm needs a larger neural network structure to fully extract its features, resulting in the increase in the training time. The irrelevant information selected will interfere with the training process and even lead to training failure. As presented in the previous chapter, state information $s_{t}=\left(x_{t},v_{t},o_{t}\right)\in S^{34}$ includes the position information $x_{t}$ of the target point and AUV, the actual velocity information $v_{t}$ of AUV, and the obstacle information $o_{t}$ detected through the obstacle avoidance sonar.

First, $x_{t}=\left(x_{d},x_{r}\right)\in R^{3}$ should include not only the distance information $x_{d}$ between the AUV and the target point, but also their relative position information $x_{r}$. $x_{d}$ is helpful for the AUV to approach the target point. In the experimental process of this research, the position of the target point is random; hence, it is not enough to obtain the distance between them. The position information $x_{r}$ of the AUV and the target point in the world coordinate system must also be obtained.

(14) $$x_{r}=\left(x_{goal}-x_{AUV},y_{goal}-y_{AUV}\right)\in R^{2},$$

where $\left(x_{AUV},y_{AUV}\right)$ and $\left(x_{goal},y_{goal}\right)$ represent the coordinates of the AUV and the target point in the world coordinate system, respectively. According to the AUV motion planning task, we do not need to know all the position information of either of them. We just need to know the relative position of the AUV and the target point. Thus, $x_{t}\in R^{3}$.

Second, the velocity information of the AUV $v_{t}=\left(v_{s},v_{\psi }\right)\in R^{4}$ should include the size of the AUV speed $v_{s}$ and the direction of the movement $v_{\psi }$. In the maneuverability equation of the AUV, the trajectory planned by AUV is greatly related to its own speed, which is introduced into the neural network as an observation vector. $v_{s}$ can be obtained by DVL combined with the inertial navigation system. $v_{s}=\left(u,v,r\right)\in R^{3}$. However, this factor only includes the magnitude of the velocity. The direction of the velocity will also affect the success of the AUV motion planning task. A vector $\left(x,y\right)$ to represent the direction of the surge velocity u requires two values, which increases the dimension of the state space; hence, it can be expressed by the following formula:

(15) $$v_{\psi }=Angle\left(u,x_{r}\right),$$

where u represents the vector of the surge velocity of the AUV, $x_{r}$ represents the vector of the AUV position pointing to the target position, and $Angle\left(u,x_{r}\right)$ denotes the angle between two vectors. Based on this method, a numerical value can be used to express the direction of the velocity, and $v_{\psi }$ can train the ability of the AUV to point to the target point in the speed direction and reduce the distance of the trajectory.

**Figure 3:**
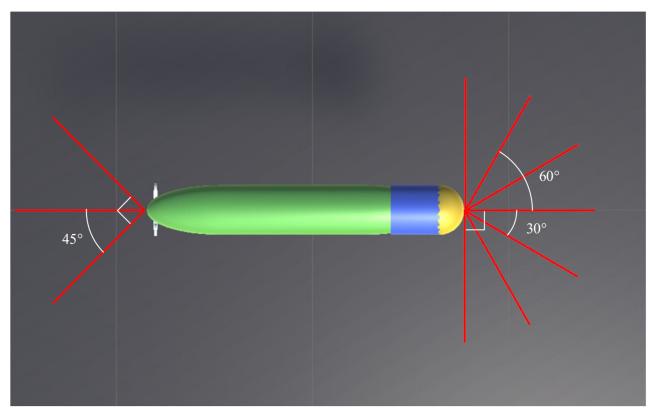
AUV sonar model.

Finally, an obstacle avoidance sonar must be used to obtain obstacle position information $o_{t}$ to help AUV avoid obstacles in real time. The maximum detection distance of sonar is 20 m. Figure 3 shows the AUV sonar model. The AUV is equipped with a total of 10 obstacle avoidance sonars; accordingly, it can detect obstacle distance information in ten directions; hence, $o_{t}\in R^{10}$ is a 10-dimensional state space.

In the actual process, we normalize the value of the observation vector to the range [−1, +1] or [0, 1]. This normalization processing is based on the mean value and variance of the observation vector. In this way, the convergence rate of the neural network is faster in most cases. 

AUV will inevitably encounter certain situations, such as unclear target locations, detection equipment failure, and loss of tracking targets, due to the uncertainty of the underwater environment. This situation is a big challenge for motion planning tasks. Part of the observations received by the planning system often includes incomplete information. To overcome this problem, we use a memory enhancement method. A limited “memory” can be provided to an agent via a stacked approach without the complexity of adding a recurrent neural network. Stack means repeating the observations from the previous step as a larger observation vector as input to the neural network. For example, when the agent performs four steps, Table 4 shows the effect of stack:

In this way, the neural network can compare the behavior of the AUV and the changes in the reward value in several observations before and after. Accordingly, the neural network can better extract the characteristics of the observation vector. For example, the size of the stack in this experiment is set to two; hence, the state space of the AUV in this research is 17 × 2 = 34 dimensions, and the observed vector values available are also 34. The neural network can better update its parameters and achieve the training goal through the difference of the reward value when the information, such as the distance difference and speed difference between the two steps before and after the AUV, changes. However, the size of the stack should be limited to a certain range according to the different tasks of its own, because a simple change of its value will cause the dimension of the observation vector to exponentially increase or decrease, which will affect the training result.

The two types of action space are discrete and continuous. The discrete action space simplifies the AUV’s motion model, which can effectively reduce the difficulty of the task and improve the efficiency of exploration. The training time of the continuous motion space is longer, but the convergence effect is better. The planned trajectory is smoother in the robot motion planning task, and it is closer to its real motion state than discrete motions. However, the probability of each action cannot be directly calculated because of the infinite actions. We can only obtain the probability distribution of actions in a certain action space. Next, we want to parameterize a policy that expresses the distribution of actions. The most commonly used distribution is the Gaussian distribution:

(16) $$\pi \left(a|s,f\right)=\frac{1}{\sigma \left(s,f\right)\sqrt{2\pi }}\exp\left(-\frac{{\left(a-\mu \left(s,f\right)\right)}^{2}}{2\sigma {\left(s,f\right)}^{2}}\right).$$

According to Equation (11), the external force output by the propeller only includes the surge force $\tau_{u}$ and yaw moment $\tau_{r}$ and does not include the transverse thrust. These factors can control the surge velocity and yaw angular velocity of AUV and change its trajectory. Therefore, the motion space of the motion planning problem described in this research is a 2D motion space. In this research, the output value of the neural network is controlled between (−1,1), and a simple linear transformation is carried out:

(17) $$\tau_{u}=clip\left(-1,1\right)\times 20_{1}+10,$$

(18) $$\tau_{r}=clip\left(-1,1\right)\times 30.$$

The action vectors $\tau_{u}\in \left(-10,30\right)$ and $\tau_{r}\in \left(-30,30\right)$ are two floating-point numbers, the sign indicates the direction of force, and the absolute value denotes the magnitude of force and moment. The purpose of the transformation is to select an appropriate action boundary based on the actual physical model, avoid missing actions, and remove irrelevant actions. The purpose of $\tau_{u}\in \left(-10,30\right)$ is to impose a certain limit on the deceleration on the action output and reduce the movement state of the AUV backward.

### 3.2. SAC

SAC is an off-policy actor–critic model following the reinforcement learning framework. This model incorporates the entropy measure of the policy into the reward to encourage exploration. The purpose of training is not only to maximize the sum of expected returns but also to maximize the entropy of the policy. The objective function [49] is defined as follows:

(19) $$J\left(\pi \right)=\sum_{t=0}^{T}E_{\left(s_{t},a_{t}\right)∼\rho_{\pi }}\left[r\left(s_{t},a_{t}\right)+\alpha H\left(\pi \left(\cdot |s_{t}\right)\right)\right],$$

(20) $$H\left(\pi \left(\cdot |s_{t}\right)\right)=-\sum_{a_{t}}\pi \left(a_{t}|s_{t}\right)\log\pi \left(a_{t}|s_{t}\right),$$

where *π* is the policy, $H\left(\pi \left(\cdot |s_{t}\right)\right)$ is the entropy term, and α denotes the temperature parameter, which can control the proportion of this entropy term in the total reward. The larger the value of α, the stronger the randomness of the strategy. $\rho_{\pi }\left(s\right)$ and $\rho_{\pi }\left(s,a\right)$ denote the state and state-action marginals of the state distribution induced by the policy $\pi \left(a_{t}|s_{t}\right)$.

According to the soft Bellman equation [50,51], the soft action value function $Q_{soft}\left(s_{t},a_{t}\right)$ and soft state value function $V_{soft}(s_{t})$ are defined as follows:

(21) $$Q_{soft}\left(s_{t},a_{t}\right)=r\left(s_{t},a_{t}\right)+\gamma E_{s_{t+1}∼\rho }\left[V_{soft}\left(s_{t+1}\right)\right].$$

(22) $$V_{soft}(s_{t})=E_{a_{t}∼\pi }\left[Q_{soft}\left(s_{t},a_{t}\right)-\alpha \log\pi \left(a_{t}|s_{t}\right)\right].$$

SAC is committed to learning three functions:

1. $\pi_{\phi }\left(s_{t}|a_{t}\right)$ with the neural network parameter ϕ
2. Soft action value function $Q_{\theta }\left(s_{t},a_{t}\right)$ with the neural network parameter θ
3. Soft state value function $V_{\psi }$ parameterized by ψ. In principle, there is no need to set a separate function approximator for the state value, because $V_{\psi }$ can be derived from *Q* and π, according to Equation (22).

The soft Q-function is trained to minimize the soft Bellman residual:

(23) $$J_{Q}\left(\theta \right)=E_{\left(s_{t},a_{t}\right)∼D}[\frac{1}{2}{\left(Q_{\theta }\left(s_{t},a_{t}\right)-\left(r\left(s_{t},a_{t}\right)+\gamma E_{s_{t+1}∼\rho }\left[V_{\bar{\theta }}\left(s_{t+1}\right)\right]\right)\right)}^{2}],$$

where *D* denotes the replay buffer. According to Equation (22), $V_{\bar{\theta }}$ can be replaced by $Q_{\bar{\theta }}\left(s_{t+1},a_{t+1}\right)$, and Equation (23) is optimized by stochastic gradients:

(24) $${\hat{\nabla }}_{\theta }J_{Q_{\theta }}=\nabla_{\theta }Q_{\theta }\left(s_{t},a_{t}\right)\left[Q_{\theta }\left(s_{t},a_{t}\right)-\left(r\left(s_{t},a_{t}\right)+\gamma \left(Q_{\bar{\theta }}\left(s_{t+1},a_{t+1}\right)-\alpha \log\left(\pi_{\phi }\left(a_{t+1}|s_{t+1}\right)\right)\right)\right)\right],$$

where target soft Q-function with parameters $\bar{\theta }$ is obtained from θ through exponentially moving average (EMA). The specific update method of $\bar{\theta }$ is as follows:

(25) $$\bar{\theta }=\tau \theta +\left(1-\tau \right)\bar{\theta },$$

where $\tau \in \left[0,1\right]$ determines the magnitude of the update.

The objective function of the policy network is to minimize the KL-divergence between two distributions:

(26) $$\begin{array}{ll} J_{\pi }\left(\phi \right) & =D_{KL}\left(\pi_{\phi }\left(\cdot |s_{t}\right)∥\exp\left(\frac{1}{\alpha }Q_{\theta }\left(s_{t},\cdot \right)-\log Z\left(s_{t}\right)\right)\right)\\ & {=E}_{s_{t}∼D,a_{t}∼\pi_{\phi }}\left[\log\left(\frac{\pi_{\phi }\left(a_{t}|s_{t}\right)}{\exp\left(\frac{1}{\alpha }Q_{\theta }\left(s_{t},\cdot \right)-\log Z\left(s_{t}\right)\right)}\right)\right]\\ & =E_{s_{t}∼D,a_{t}∼\pi_{\phi }}\left[\log\pi_{\phi }\left(a_{t}|s_{t}\right)-\frac{1}{\alpha }Q_{\theta }\left(s_{t},a_{t}\right)+\log Z\left(s_{t}\right)\right] \end{array},$$

where $Z\left(s_{t}\right)$ denotes the partition function. Then, we need to obtain the gradient of $J_{\pi }\left(\phi \right)$. However, Equation (26) needs to be simplified before this. We can get (multiplied by α and ignore the log-partition function $\log Z\left(s_{t}\right)$, because it does not affect the gradient of $J_{\pi }\left(\phi \right)$ to ϕ:

(27) $$J_{\pi }\left(\phi \right)=E_{s_{t}∼D}\left[E_{s_{t},a_{t}∼D}\left(\alpha \log\pi_{\phi }\left(a_{t}|s_{t}\right)-Q_{\theta }\left(s_{t},a_{t}\right)\right)\right],$$

where $\pi_{\phi }\left(a_{t}|s_{t}\right)$ is not differentiable. Hence, we use the reparameterization trick to get the action here.

(28) $$a_{t}=f_{\phi }\left(\varepsilon_{t};s_{t}\right)=f_{\phi }^{u}\left(s_{t}\right)+\varepsilon_{t}⊙f_{\phi }^{\sigma }\left(s_{t}\right).$$

The f function outputs the mean and variance, and the ε is noise, sampling from the standard Gaussian distribution. The whole process is completely differentiable using this trick. Equation (27) can be rewritten as follows:

(29) $$J_{\pi }\left(\phi \right)=E_{s_{t}∼D,\varepsilon_{t}∼N}\left[\alpha \log\pi_{\phi }\left(f_{\phi }\left(\varepsilon_{t};s_{t}\right)|s_{t}\right)-Q_{\theta }\left(s_{t},f_{\phi }\left(\varepsilon_{t};s_{t}\right)\right)\right].$$

Then, the gradient of $J_{\pi }\left(\phi \right)$ is:

(30) $${\hat{\nabla }}_{\theta }J_{\pi }\left(\phi \right)=\nabla_{\phi }\alpha \log\left(\pi_{\phi }\left(a_{t}|s_{t}\right)\right)+\left(\nabla_{a_{t}}\alpha \log\pi_{\phi }\left(a_{t}|s_{t}\right)-\nabla_{a_{t}}Q\left(s_{t},a_{t}\right)\right)\nabla_{\phi }f_{\phi }\left(\varepsilon_{t};s_{t}\right).$$

In most cases, temperature parameter α, which determines the success of training, is difficult to set. However, using a fixed α is unreasonable due to the constant change in reward, which will make the whole training unstable and lose the advantage of SAC’s low dependence on hyperparameters. Therefore, we hope that the neural network can automatically adjust the size of α to ensure that it can be adjusted to different values in various states. When an AUV explores a new area, α is increased to encourage it to explore more possibilities. When an optimal policy has been achieved in a certain state, α is reduced to gradually converge.

**Theorem** **1.** *We can use entropy as a constraint to solve the optimization problem of policy and α:*

(31) $$\underset{\pi_{0}:T}{\max}E_{\rho_{\pi }}\left[\sum_{t=0}^{T}r\left(s_{t},a_{t}\right)\right]\;s.t.\forall t,H\left(\pi_{t}\right)\geq H_{0},$$

*where $H_{0}$ is a minimum entropy threshold. Based on the above constrained optimization problem, the objective function of temperature parameter α can be obtained as follows:*

(32) $$J\left(\alpha \right)=E_{a_{t∼}\pi_{t}}\left[-\alpha \log\pi_{t}\left(a_{t}|s_{t}\right)-\alpha H_{0}\right].$$

**Proof of Theorem** **1.** See Appendix A. □

We will use stochastic gradient descent methods to ensure that the system will automatically select the appropriate temperature coefficient α to keep the entropy of the policy in a dynamic balance suitable for training.

**Figure 4:**
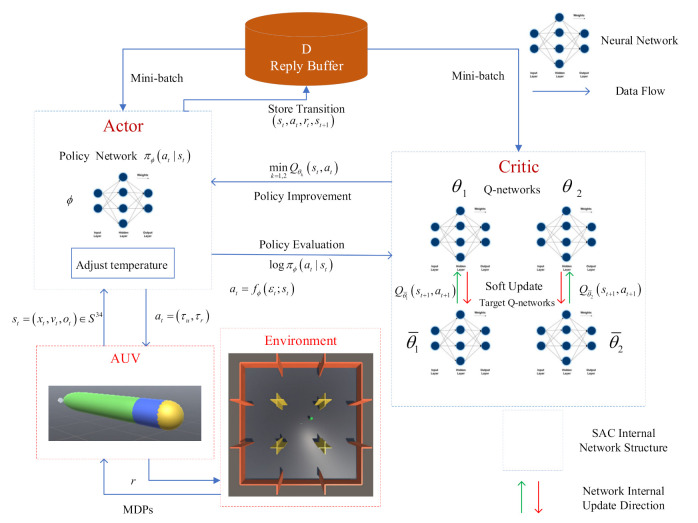
Neural network structure of the SAC algorithm.

Figure 4 shows the neural network structure of the SAC algorithm designed in this research. The structure is the same as the general actor–critic framework. The actor is responsible for executing decisions. Meanwhile, the critic is responsible for guiding whether the actor’s decision is correct. The actor is composed of a policy network, and the mean and variance of the Gaussian distribution are outputted in the actual process. The critic borrows the idea of DDQN to reduce the positive bias in the policy improvement step. In the actual design process, two soft Q-function networks are used, with parameters $\theta_{1}$ and $\theta_{2}$. The minimum value $\underset{k=1,2}{\min}Q_{\theta_{k}}\left(s_{t},a_{t}\right)$ between these factors is used to train the policy network. Two target Q-function networks are used to update the Q-function network, and their own parameters, ${\bar{\theta }}_{1,2}$, are updated using Equation (25) in a small amount. In addition to training the soft Q function and policy, we also learn α by minimizing the objective function in Equation (32). Afterwards, the experience is collected through the continuous interaction between the AUV and the environment, the transition $\left(s_{t},a_{t},r_{t},s_{t+1}\right)$ is stored in the replay buffer D, and mini-batch experience is sampled from the replay buffer each time. Finally, we use the method of stochastic gradient descent to train the neural network parameters.

The algorithm flow of this article based on SAC shown in Algorithm 1.
**Algorithm 1: SAC**1: **Input:**
$\theta_{1}$, $\theta_{2}$, ϕ⊳Initial parameters2: ${\bar{\theta }}_{1}\leftarrow \theta_{1}$, ${\bar{\theta }}_{2}\leftarrow \theta_{2}$⊳Initialize target network weights3: D←∅⊳Initialize replay buffer4:   **for** each iteration **do**
5:     **for** each environment step **do**
6:       $a_{t}∼\pi_{f}\left(a_{t}|s_{t}\right)$⊳Sample action from the policy7:       $s_{t+1}∼p\left(s_{t+1},s_{t},a_{t}\right)$⊳Sample transition from the environment8:       $D\leftarrow D\cup \{\left(s_{t},a_{t},r_{t},s_{t+1}\right)\}$⊳Store the transition in the replay buffer9:     **end for**
10:     **for** each gradient step **do**
11:       $\theta_{i}\leftarrow \theta_{i}-\lambda_{Q}{\hat{\nabla }}_{\theta_{i}}J_{Q}\left(\theta_{i}\right)\;for\;i\in \{1,2\}$⊳Update the Q-function parameters12:       $\phi \leftarrow \phi -\lambda_{\pi }{\hat{\nabla }}_{f}J_{\pi }\left(\phi \right)$⊳Update policy weights13:       $\alpha \leftarrow \alpha -\lambda {\hat{\nabla }}_{\alpha }J\left(\alpha \right)$⊳Adjust temperature14:       ${\bar{\theta }}_{i}=\tau \theta_{i}+\left(1-\tau \right){\bar{\theta }}_{i}\;for\;i\in \{1,2\}$⊳Update target network weights15:     **end for**16:   **end for**17: **Output:**
$\theta_{1}$, $\theta_{2}$, ϕ⊳Optimized parameters

### 3.3. GAIL

A problem is often encountered in the process of using reinforcement learning to train agents. In some environments with sparse rewards or only end rewards, the initial reward in the environment for the policy obtained by the random initialization of the neural network is difficult to obtain. Therefore, a good solution is to obtain a pre-trained neural network with expert characteristics through IL to help the agent better explore the environment. In most cases, IL can be used to obtain a policy from behavioral data generated by the expert. A common method of IL is based on IRL, which first learns the cost function of expert data and uses RL methods to learn the expert policy. 

The objective function of maximum entropy inverse reinforcement learning is introduced [52]:

(33) $$IRL_{\psi }\left(\pi_{E}\right)=\underset{c\in C}{\arg\max}-\psi \left(c\right)+\left(\underset{\pi \in \Pi }{\min}-H\left(\pi \right)+E_{\pi }c\left(s,a\right)\right)-E_{\pi_{E}}\left[c\left(s,a\right)\right].$$

The problem is not fully constrained because many policies may lead to the same trajectory; hence, the entropy term $H\left(\pi \right)$ is added to the original IRL formula. c∈C:S×A→R denotes the cost function. ψ:C→R is the regularizer of the cost function, which will assign a value to each cost function c to solve the problem of easy overfitting under small data sets. ∏ contains all stationary stochastic policies that take actions in A given states in S. $\pi_{E}$ is the expert policy. The output here is the desired cost function. The second step of this framework is to input the learned cost function into a standard reinforcement learning problem. We use the state–action occupancy distribution ρ to express the above the problem. $\rho_{\pi }\left(s,a\right)$ represents the state–action distribution encountered when the agent uses the policy to interact with the environment. 

Define $\rho_{\pi }\left(s,a\right)=\pi \left(a|s\right)\sum_{t=0}^{\infty }\gamma^{t}P\left(s_{t}=s|\pi \right)$, where $s_{t+1}∼P\left(s_{t+1}|s_{t},a_{t}\right)$.

For any cost function *c*,

(34) $$E_{\pi }\left[c\left(s,a\right)\right]=E\left(\sum_{s,a}\rho_{\pi }\left(s,a\right)c\left(s,a\right)\right).$$

Then, an entropy-regularized version of *RL* can be described as follows:

(35) $$RL\circ IRL_{\psi }\left(\pi_{E}\right)=\underset{\pi \in \Pi }{\arg\min}-H\left(\pi \right)+\psi^{*}\left(\rho_{\pi }-\rho_{\pi_{E}}\right),$$

where $\psi^{*}\left(\rho_{\pi }-\rho_{\pi_{E}}\right)=\sup_{c\in C}{\left(\rho_{\pi }-\rho_{\pi_{E}}\right)}^{T}c-\psi \left(c\right)$ denotes the convex conjugate of ψ, which represents a measure of similarity between the occupancy measures of the expert policy $\pi_{E}$ and the agent policy π. The purpose of training is achieved by maximizing entropy and minimizing the occupancy distribution of the two policies.

Different regularizers $\psi \left(c\right)$ represent various IL algorithms. We define GAIL as follows [41]:

(36) $$\psi_{GA}\left(c\right)≜\left\{\begin{array}{ll} E_{\pi_{E}}\left[g\left(c\left(s,a\right)\right)\right] & if\;c<0\\ +\infty & otherwise \end{array}\right.,\;where\;g\left(x\right)=\left\{\begin{array}{ll} -x-\log\left(1-e^{x}\right) & if\;x<0\\ +\infty & otherwise \end{array}\right.,$$

where $\psi_{GA}$ is derived from the idea of binary classification. The regularizer has a lower penalty to the cost function c, which assigns a certain amount of negative cost to the expert state action pairs. However, if c allocates large costs (close to zero) to experts, then $\psi_{GA}$ will severely punish.

Then, we can obtain:

(37) $$\psi_{GA}^{*}\left(\rho_{\pi }-\rho_{\pi_{E}}\right)=\underset{D\in {\left(0,1\right)}^{S\times A}}{\max}E_{\pi }\left[\log D\left(s,a\right)\right]+E_{\pi_{E}}\left[\log\left(1-D\left(s,a\right)\right)\right],$$

where $D:S\times A\to \left(0,1\right)$ denotes the discriminator. Equation (37) is equivalent to using a negative log loss function to distinguish between π and $\pi_{E}$. The expression of the convex conjugate of the cost regularizer is substituted into objective Equation (37). We can obtain the objective function of GAIL [53]:

(38) $$\underset{\pi \in \prod }{\min}\underset{D\in \left(0,1\right)}{\max}E_{\pi }\left[\log D\left(s,a\right)\right]+E_{\pi_{E}}\left[\log\left(1-D\left(s,a\right)\right)\right]-\lambda H\left(\pi \right).$$

The optimal loss in Equation (37) is equal to the Jensen–Shannon divergence of two distributions, namely,

(39) $$\psi_{GA}^{*}\left(\rho_{\pi }-\rho_{\pi_{E}}\right)=D_{JS}\left(\rho_{\pi },\rho_{\pi_{E}}\right)≜D_{KL}\left(\rho_{\pi }||\left(\rho_{\pi }+\rho_{\pi_{E}}\right)/2\right)+D_{KL}\left(\rho_{\pi_{E}}||\left(\rho_{\pi }+\rho_{\pi_{E}}\right)/2\right).$$

Then, we can obtain:

(40) $$\underset{\pi }{\mathrm{minimize}}\psi_{GA}^{*}\left(\rho_{\pi }-\rho_{\pi_{E}}\right)-\lambda H\left(\pi \right)=D_{JS}\left(\rho_{\pi },\rho_{\pi_{E}}\right)-\lambda H\left(\pi \right).$$

**Figure 5:**
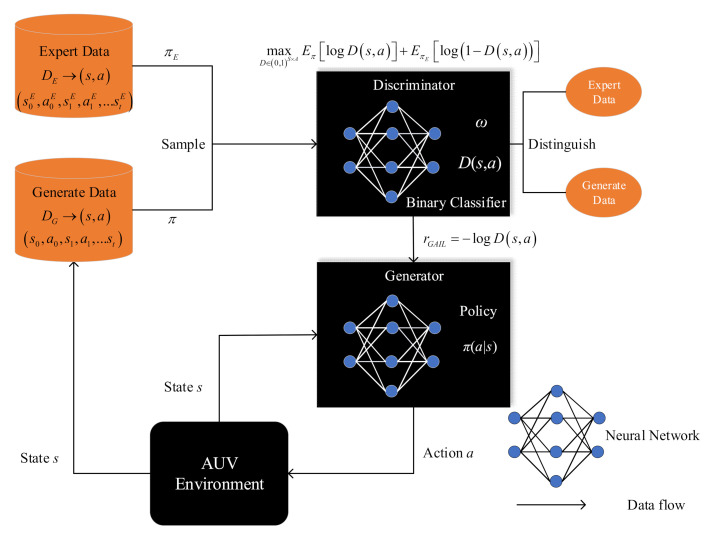
Neural network structure of the GAIL algorithm.

Figure 5 shows the neural network structure of GAIL. GAIL’s network settings refer to the idea of GAN, and the structure includes a generator G, which is the policy network here, denoted by $\pi_{\phi }\left(a|s\right)$, with weights ϕ. The structure also contains a discriminator $D_{\omega }$ with weights ω. The discriminator tries to distinguish whether the samples are generated by experts or by the policy π. We use an Adam gradient algorithm to make Equation (38) increase for ω. The purpose of generator G is to confuse discriminant classifier D to ensure that it cannot distinguish whether the trajectory is from the expert or policy. Specifically, the Jensen–Shannon divergence between the occupancy measure of the policy $\rho_{\pi }$ and the occupancy measure of the expert policy $\rho_{\pi_{E}}$ is minimized. The discriminator can be used to obtain a reward function, which, in our case, is simply $r_{GAIL}=-\log D\left(s,a\right)$. In the original GAIL algorithm, we use trust region policy optimization (TRPO) to update the policy π with the internal reward function.

In this research, the SAC algorithm is used in the policy network, and GAIL plays an auxiliary role in training. When the expert samples in the demonstration are ineffective, the optimal policy cannot be obtained by only relying on the method of IL. The idea of maximum entropy in GAIL is consistent with the purpose of SAC and can be combined well. Furthermore, SAC is off-policy, which improves the sample efficiency compared with the original GAIL algorithm using TRPO to train the network. The combination of GAIL and SAC enhances the agent’s exploration performance compared with using the GAIL algorithm alone, and GAIL can assist it in accelerating training. However, GAIL introduces a survivor bias in the learning process, which encourages the agent to live as long as possible by giving positive rewards based on the similarity with the expert. This notion is in direct conflict with goal-oriented tasks (such as the AUV motion planning task in this research, encouraging the AUV to reach the target point as soon as possible, thereby ending the episode as quickly as possible). In this case, we should use low strength GAIL reward signals when the AUV completes the task and guide the learning process of the AUV by mixing the GAIL reward signal and the external reward signal. In this way, the GAIL reward signal will guide the AUV in navigating until it finds the reward value signal of the external environment.

### 3.4. Reward Function

In this research, a motion planning algorithm based on maximum entropy deep reinforcement learning is proposed to solve the motion planning problem of an underactuated AUV in an unknown environment. The reward function is a key component of the planning system, which implicitly specifies the goal of the task to be solved. This function serves as the only feedback system using a scalar signal to evaluate the performance of the control behavior. In comparison with the supervised learning algorithm, the signal is more evaluative than guiding [54]. The setting of the reward function is related to the quality of the execution strategy and determines the speed and degree of the convergence of the reinforcement learning algorithm. 

**Figure 6:**
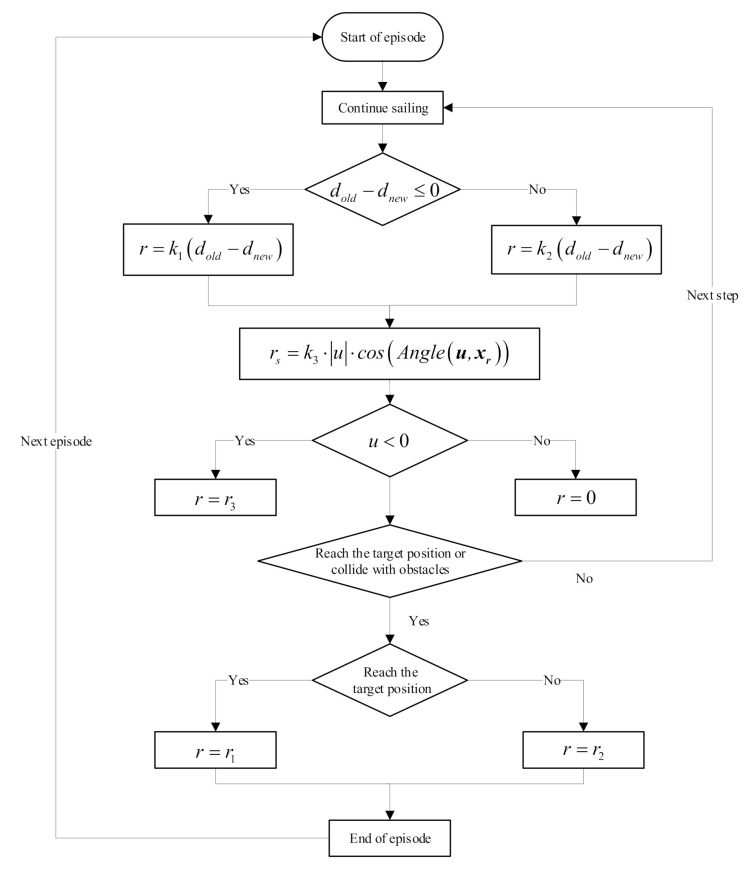
Flow chart of obtaining rewards.

In this research, the flow chart of obtaining rewards is shown in Figure 6. When the AUV reaches the target point, it achieves a positive reward value, and the episode ends; when the AUV collides with an obstacle, it achieves a negative reward value, and the episode ends. These rewards are regarded as terminal reward item $r_{end}$:

(41) $$r_{end}=\left\{\begin{array}{cc} r_{1} & if\;achieve\;goal\\ r_{2} & if\;collision \end{array}\right..$$

In other cases, the AUV continues to move. However, the motion planning itself is a task with sparse reward value because the number of times the target point can be reached is only a handful in the sample. It is not enough to rely on termination reward. The task objective should be decomposed, and the design of the reward value should be closely related to the state input. The distance reward item is designed as follows to encourage the AUV to approach the target point:

(42) $$r_{dis\tan ce}=\left\{\begin{array}{ll} k_{1}\left(d_{old}-d_{new}\right) & if\;d_{old}-d_{new}\leq 0\\ k_{2}\left(d_{old}-d_{new}\right) & if\;d_{old}-d_{new}>0 \end{array}\right.,$$

where $d_{old}$ and $d_{new}$ represent the distance between the AUV and the target point at the last and the current times, respectively; and $k_{1}$ and $k_{2}$ denote the weight of the reward value, respectively. When the distance between the last time and the target point is greater than the current time, the AUV is constantly close to the target point and will receive a positive reward; otherwise, it will receive punishment. During the experiment, $k_{1}>k_{2}$, which is used to avoid abnormal behavior of AUV in the training process. If $k_{1}\leq k_{2}$, then the agent will become “greedy”. The best profitable choice for AUV is not to arrive at the target position as soon as possible, but to constantly repeat the “close-away” actions. In this way, the profit far exceeds that of directly reaching the target point. Therefore, the purpose of $k_{1}>k_{2}$ is to urge the AUV to reach the target point as soon as possible.

The AUV motion planning task must satisfy not only the goal of completeness but also the constraints of optimality. When the task of reaching the target point and avoiding obstacles is completed, the distance s should be as short as possible, or the time t is as short as possible, and the movement trajectory is smoother. However, making all these factors in the actual process optimal is difficult due to the constraints in environmental conditions and considering the factors of AUV system dynamics. Therefore, the motion state of AUV is constrained as follows: we hope that, when there is no obstacle in front of the AUV, the heading direction of the AUV can point to the target point and sail along a straight line. When the sailing direction is accurate and fixed, the velocity u can be as large as possible to ensure that it can reach the target point quickly. Therefore, the reward items related to surge velocity u and heading angle are set as follows:

(43) $$r_{s}=k_{3}\cdot \left|u\right|\cdot \cos\left(Angle\left(u,x_{r}\right)\right),$$

where $k_{3}$ is the weight of reward value, and $Angle\left(u,x_{r}\right)\in \left[0,\pi \right]$ denotes the angle between u and $x_{r}$. The definitions of u and $x_{r}$ are the same as the state space. When $Angle\left(u,x_{r}\right)\in \left[0,\frac{\pi }{2}\right]$, $r_{s}\geq 0$. When the angle is zero, i.e., the direction of surge velocity of AUV points to the target point, the reward is the largest and increases with the increase in surge velocity *u*. When $Angle\left(u,x_{r}\right)\in \left(\frac{\pi }{2},\pi \right]$, $r_{s}<0$. Specifically, the AUV will be punished when it is far from the target point.

Performing a backward movement for a long time is discouraged in the actual planning process of AUV. Given the difficulty of control of AUV, high navigation resistance, and low propeller efficiency at this time, a penalty item must be set to limit the form of movement. However, a short-term backward process can sometimes increase the flexibility of the AUV and make its navigation route shorter; hence, the value of this reward item should be coordinated with the above problems, not only to prevent the AUV from retreating for a long time, but also to avoid losing this state of motion. Therefore, reward item $r_{u}$ limited by velocity u is expressed as follows:

(44) $$r_{u}=r_{3}\;if\;u<0.$$

Accordingly, the total reward function is set as follows:

(45) $$r=\left\{\begin{array}{cc} r_{1} & if\;achieve\;goal\\ r_{2} & if\;collision\\ k_{1}\left(d_{old}-d_{new}\right) & if\;d_{old}-d_{new}\leq 0\\ k_{2}\left(d_{old}-d_{new}\right) & if\;d_{old}-d_{new}>0\\ k_{3}\cdot \left|u\right|\cdot \cos\left(Angle\left(u,x_{r}\right)\right) & every\;step\\ r_{3} & if\;u<0 \end{array}\right..$$

## 4. Simulation and Results

In this section, simulation experiments are conducted to verify the effectiveness of the end-to-end AUV motion planning system, designed, in this research, based on the above AUV model and the proposed GAIL-SAC algorithm. Unity software is used for visual simulation, the program is written based on C # and python languages, the neural network is built by torch, and the model is trained by GPU. The experiment uses a NVIDIA Titan V graphics card with i9-7980XE processor and 32 GB of RAM.

**Figure 7:**
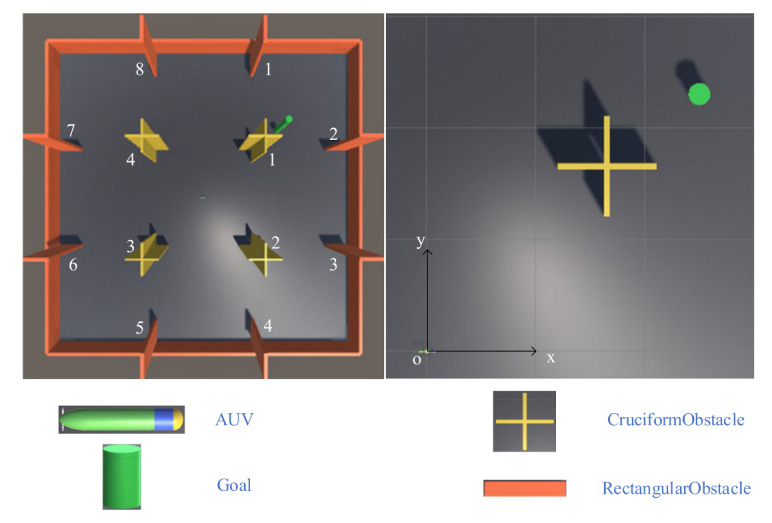
Experimental environment.

Figure 7 shows the experimental environment of the AUV. The environment size is set to 100 × 100 m, and the origin of the coordinate system is at the geometric center of the map. The motion planning task requires the AUV to reach the position of the green cylinder, which is the target position, while avoiding yellow and orange obstacles during the navigation. In comparison with other regular-shaped obstacles, such as rectangles, cross-shaped obstacles’ shapes and sizes are more difficult for sonar to detect in unknown environments. Therefore, this aspect is a challenge to the intelligent level of AUV.

During the training, the positions of the AUV and the target point are reset at the beginning of each episode. The initial position of the AUV is always at the origin of the coordinate system and the heading points to the positive direction of the x-axis. To avoid such a scenario, the AUV only learns the policy in a single environment, but it can complete the task in a variety of environments. The position of the target point is not fixed, but randomly appears on a circle with a radius of 35 centered on the origin of the coordinates. In addition to the termination of the episode caused by the AUV reaching the target point or colliding with obstacles, the experiment also limits the maximum number of training steps per episode to speed up the training and avoid the AUV from falling into a dead zone in a local position. When this value is exceeded, the episode will also end. Table 5 shows the position and shape size (collision size) of each element in the environment. The vertical position coordinates and height of each object in the environment are ignored because this research only considers the motion of the AUV in the plane.

After considerable experimental adjustment, the parameter settings of the reward value are shown in Table 6.

In this research, three methods, namely, PPO, SAC, and SAC-GAIL, are used for experimental comparison to explore the effect of AUV’s motion planning under their guidance. The specific experimental parameter are shown in Table 7.

**Figure 8:**
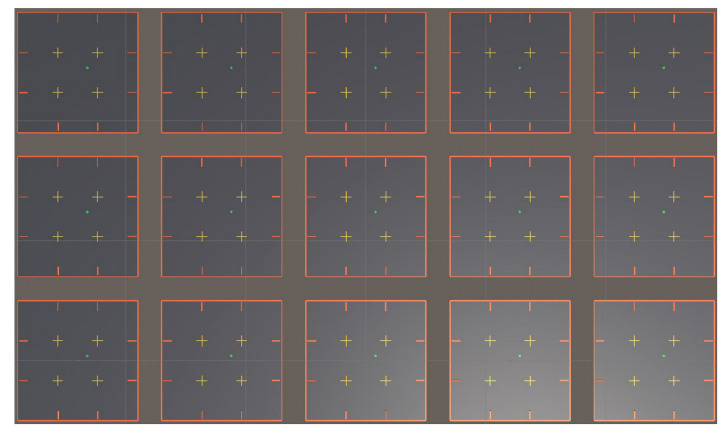
Real training environment in the Unity interface.

Multiple identical environments are present in the Unity interface in the actual training process (Figure 8). In these environments, each AUV is an independent agent, but it is equipped with the same planning system. The purpose of this condition is to speed up the training. The 15 AUVs in the scene participate in the training and share the training results, which exponentially saves the training time. Nonetheless, we cannot indefinitely copy the scene because it will take up a considerable amount of computer memory. Finally, the whole training takes approximately 10 h.

**Figure 9:**
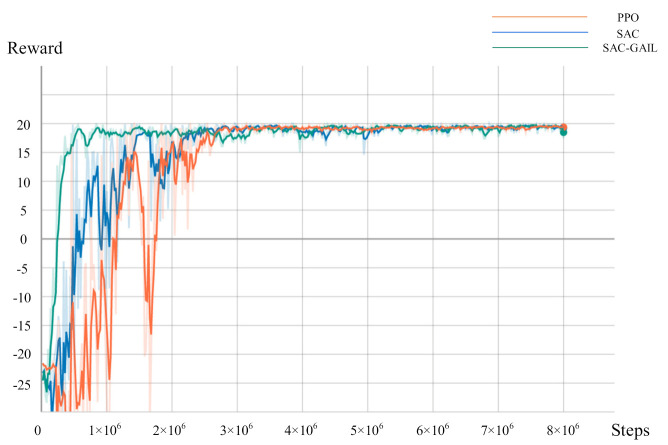
Curve of the reward value.

**Figure 10:**
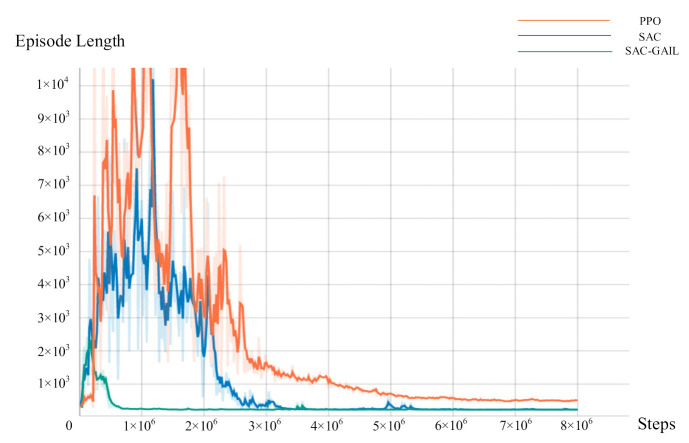
Curve of the episode length.

During the training, the Unity script is called every 0.02 s, and the AUV makes decisions every 0.1 s to select the appropriate action in the current state; hence, the time consumption per unit step of the AUV is 0.1 s. We record the mean and variance of the reward value every 20,000 steps and the number of steps required for each episode (episode length). Figure 9 and Figure 10 represent the relationship curve of the cumulative discount reward value, the episode length, and the total number of steps in the training process. The curves of three colors in the figure represent the training results of AUV motion planning under the guidance of PPO, SAC, and SAC-GAIL algorithms. In Figure 9, the reward value of the three algorithms was in a low range at the beginning of the training because AUV was still in the process of exploration at this time and did not obtain a good policy. Accordingly, the probability of collision with obstacles was high, resulting in a low reward value. Then, the curve gradually rose until it converged at approximately 20, which is consistent with the terminal reward when the AUV reaches the target point. At this time, the AUV learned an excellent policy and could successfully complete the assigned task. The curve demonstrates that the SAC-GAIL algorithm has the fastest convergence speed and ultimately reaches convergence at 100 W steps, which proves that GAIL speeds up the training process of AUV and guides the AUV route in the form of experts. SAC and PPO began to gradually converge in the 300 W steps. However, the reward value curve of the SAC algorithm rose significantly faster than that of PPO. The curve of episode length shows that the training result curves of the three algorithms all experienced a process of rising first and then falling, and the rising process represents the exploration process of AUV. The SAC-GAIL algorithm takes less exploration process and has the fastest convergence speed compared with SAC and PPO algorithms, which is consistent with the result of the reward value curve. The SAC and PPO algorithms sometimes even reached 10,000 steps in the initial stage of training. However, SAC’s episode length is generally smaller than that of the PPO algorithm. This notion means that the total number of training episodes of SAC in the case of the same total steps is higher than that of PPO, and SAC has more successful times during the training. Therefore, the exploration efficiency of AUV is higher. When the total steps of SAC reached 300 W, the episode length was stable at approximately 250, roughly the same as that of SAC-GAIL. When the total steps of PPO reached 500 W, the episode length converged to approximately 500. The episode length size means the time required for the AUV to complete the motion planning task, and the planned trajectory is longer at the same speed. Therefore, the PPO algorithm is far inferior to the SAC and SAC-GAIL algorithms in terms of time and distance optimality and does not find the optimal decision sequence.

**Figure 11:**
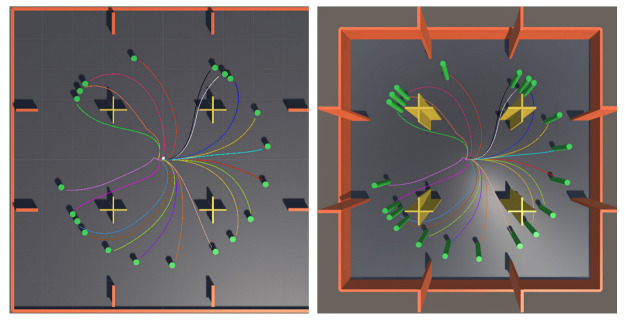
AUV motion trajectory.

After the training, the trained neural network model was saved to test the effectiveness of the motion planning system. Figure 11 shows the result of testing after the AUV model under the guidance of SAC-GAIL successfully completed training. The target position was randomly generated, and 20 experiments were successively conducted. Curves of different colors represented the actual sailing path of AUV for each time. AUV could successfully arrive at the target point from the initial position without colliding with obstacles, the trajectory was smooth, and the success rate of motion planning was 100%.

**Figure 12:**
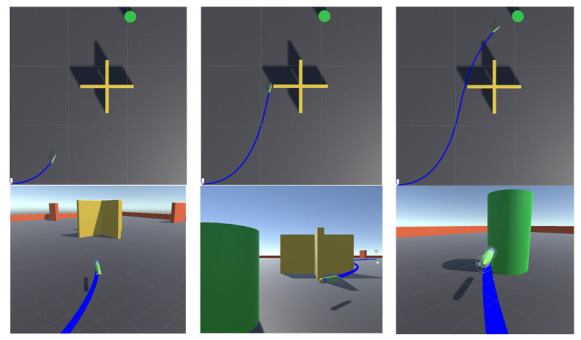
AUV local trajectory.

We select one of the experiments for a brief analysis. Figure 12 shows the local trajectory of the AUV. At this time, the position of the target point randomly appears at coordinates (20.5, 28.4), and the initial direction of the AUV is the positive direction of the x-axis. The local trajectory exhibits that the AUV accurately avoided the cross-shaped obstacles. The heading successfully pointed to the green target point when the front was unobstructed, the navigation distance was short, the trajectory was smooth, and the motion planning task was successfully completed. 

The effectiveness and completeness of the motion planning system in this work is impossible to judge with only the reward value and episode length curve in the training process. Based on the above test process, the following is to verify the differences in the route, planning time, and movement selection planned by AUV under the guidance of PPO, SAC, and SAC-GAIL. The specific rules of the test process are the same as those in the training process. The center position of the green cylinder goal is 35 m away from the AUV’s center of gravity. After considering the radius of the cylinder goal and the AUV’s own length, the shortest distance for the AUV to reach the target position is 33.27 m. 

**Figure 13:**
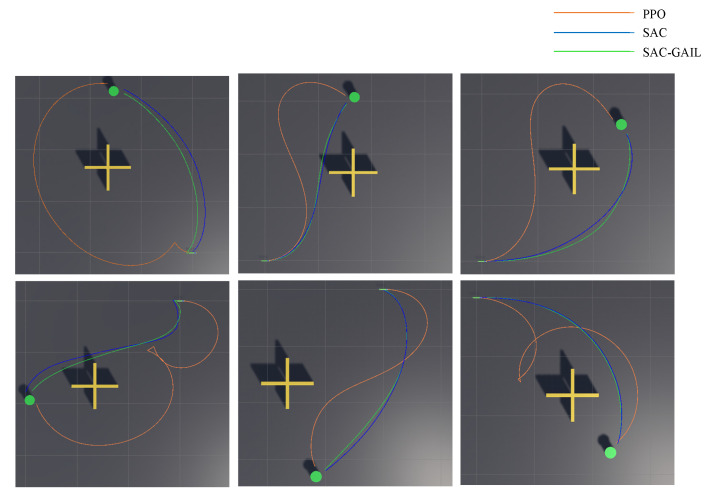
Six groups of actual planned trajectory routes.

Figure 13 shows the six groups of actual planned trajectory routes. Table 8 records the distance and time information of the trajectory planned by the three algorithms. The table illustrates that the PPO algorithm is not as good as SAC and SAC-GAIL in terms of distance or time, which can also be seen from Figure 13. During the voyage, the AUV heading angle under the guidance of the PPO algorithm more sharply changes, and the planned trajectory is not smooth enough. This difference is even more obvious when the target points randomly appear to the left of the environment. At the same time, according to the test results, it can be concluded that the difference in planning performance between SAC and SAC-GAIL is very small. Therefore, the amount of data increased, and 30 sets of experiments were carried out. In the experiment of 30 groups, GAIL performed better in 10 groups, and SAC performed better in seven groups. In the remaining 13 groups, 10 groups of data show that SAC-GAIL is better at planning distance, but the planning time is slightly worse than that of the SAC algorithm. Only three sets of data show that SAC-GAIL is better at planning time. I think the above results are mainly due to the following reasons:

1. The instability of reinforcement learning training affects the results of the experiment. Even when the number of training steps and other variables are consistent, the strategies obtained after multiple trainings are not the same, resulting in different training results. In some locations where there are more random generations and the AUV is reached earlier, the obtained strategy is more complete, and the planned route and time are better;
2. According to the reward curve and the episode length curve, SAC and SAC-GAIL have completely reached convergence for 800 W training steps; however, the convergence speed of SAC-GAIL is significantly faster. When the number of trainings is sufficient, and the reward function is properly set, the AUV can explore the terminal reward during the training process, and SAC can still play its advantages. If the total number of training steps is limited to a smaller range, the planning effect of SAC will be inferior to the SAC-GAIL algorithm;
3. The quality of the samples in the demo used in the IL will also greatly affect the effect of SAC-GAIL. The demo recorded in the model learning in this experiment is recorded on the basis of the strategy obtained after the SAC algorithm training. Hence, the planning effect is not much different from the SAC algorithm. The proportion of the GAIL signal and the external rewards will also change the total rewards received by the AUV in each episode, which will affect the strategies obtained;
4. In most cases, SAC-GAIL is superior to SAC in only one aspect of the planned trajectory distance and planning time. The reason for this phenomenon is that AUV motion planning can efficiently optimize time and distance at the same time. When the AUV sails at a higher speed, there will be a larger turning radius and a longer distance traveled per unit time. When the AUV wants to quickly reach the target point, the distance limit must be discarded, and vice versa. GAIL introduces a survivor bias in the learning process, which encourages the agent to live as long as possible by giving positive rewards based on the similarity with the expert. This notion is in direct conflict with goal-oriented tasks Therefore, the planned trajectory route based on the SAC-GAIL algorithm is shorter in most cases; however, the planning time is longer than that of the SAC algorithm.

**Figure 14:**
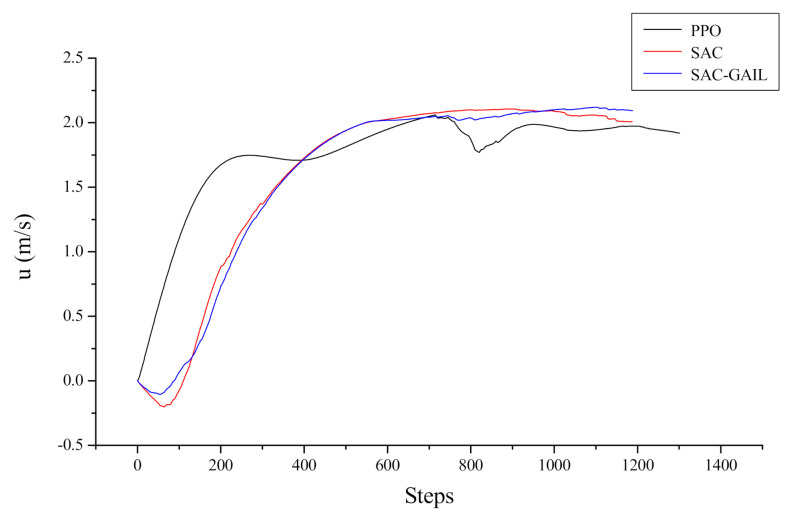
Curve of surge velocity.

**Figure 15:**
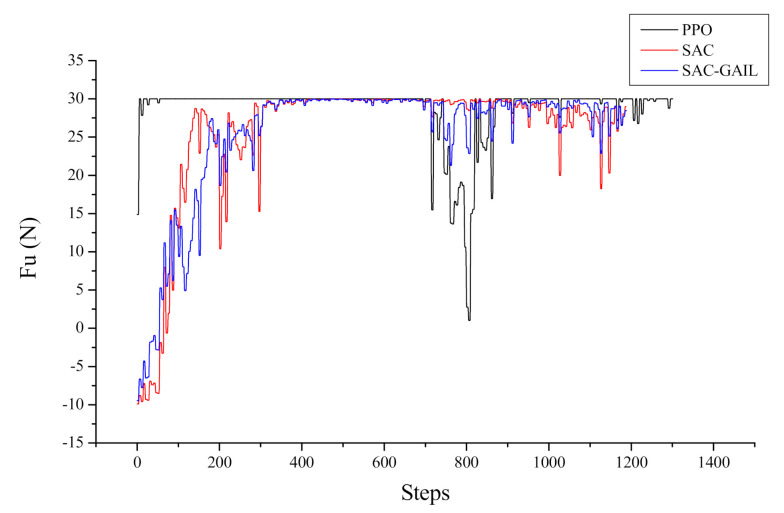
Curve of surge force.

Let us take the target point appearing at coordinates (−11.6, −33.0) as an example, corresponding to the fifth picture in Figure 13. The specific decision-making process of the AUV is analyzed from the direction of the dynamics and kinematics model. Figure 14 and Figure 15 show the variation curves of surge velocity and surge force with respect to the steps in the episode, where the unit step time is 0.02 s, and the AUV makes decisions every 0.1 s. The curves of surge velocity and force illustrate that the initial velocity of PPO $u_{0}>0$, while the other two algorithms choose to output the reverse thrust in the initial state of the AUV and perform a short backward movement, so $u_{0}<0$. We do not encourage the AUV to perform long-term backward motion, but it does not mean that this motion state is prohibited. In combination with Figure 13, this study concluded that the AUV under the guidance of the PPO algorithm traveled a larger distance to change the heading angle at the beginning of the episode. In the later stage of sailing, the velocity of AUV is stable approximately 2.0 m/s, which is in line with the AUV dynamic model. When the surge force remains unchanged, the hydrodynamic resistance of the AUV will increase with the increase in velocity, allowing the AUV to maintain a stable velocity. The surge force curve illustrates that the AUV under the guidance of PPO outputs 30 N thrust near the initial state, which is the maximum force output by the propeller specified in this research. The thrust output of the AUV under the guidance of SAC and SAC-GAIL gradually changes, which is more in line with the actual situation of the actuators in the real world. Figure 15 shows that the thrust output of the propeller is stable near the boundary action when the AUV reaches a stable sailing state, which enables the AUV to quickly reach the target position and meet the planning goal of time optimality.

**Figure 16:**
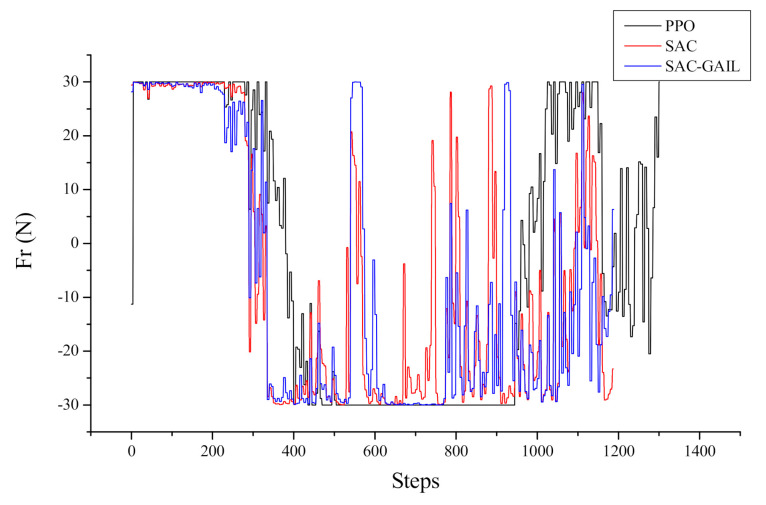
Curve of yaw moment output by AUV.

**Figure 17:**
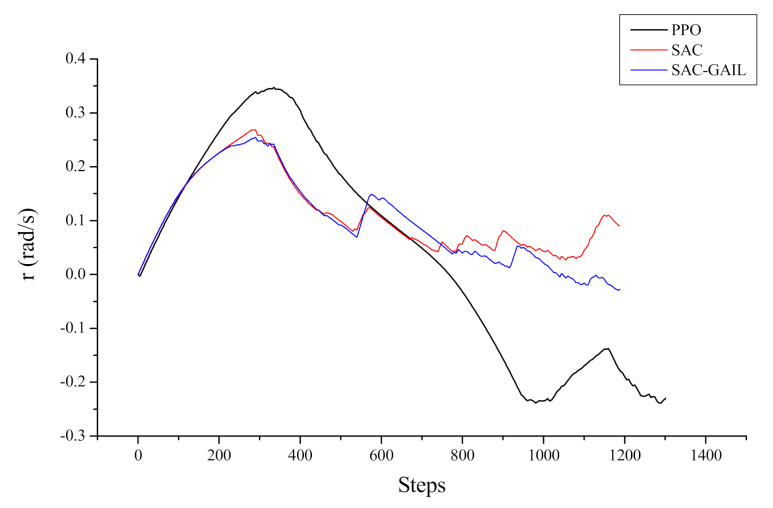
Curve of angular velocity in the yaw output by AUV.

Figure 16 and Figure 17 represent the curves of the yaw moment and angular velocity in yaw output by AUV with respect to the number of steps in the episode, respectively. The decision-making process of AUV cannot be seen directly from the yaw moment curve, and the curve is oscillating because the AUV dynamic model is complex, and the mapping from force to velocity requires a cumbersome calculation process. Therefore, the effect can be shown by the change in the angular velocity in yaw and heading angle. After 700 steps, the AUV angular velocity, under the guidance of SAC and SAC-GAIL, changed within 0.1 rad/s. This notion means that the forward direction of AUV slowly changed at this time, and the sailing trajectory was inclined to a straight line. However, after 700 steps, the AUV guided by PPO moves in the opposite direction, and the angular velocity in yaw gradually increases, which is the same as the trajectory effect in Figure 13. Accordingly, the route planned by the AUV under the guidance of PPO is “S”, while the trajectory of the AUV under the guidance of SAC and SAC-GAIL is a smooth arc and tends to a straight line in the second half of the trajectory. 

**Figure 18:**
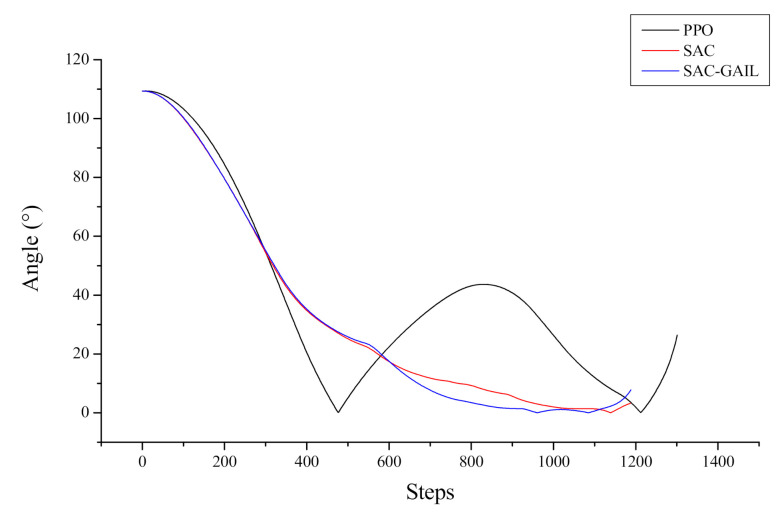
Angle curve of AUV.

In Figure 18, the curve of the change of the included angle between the navigation direction of AUV and the target position relative to the number of steps in the episode is shown. Here, the angle in the reward function is chosen to replace the heading angle in the vertical axis of the coordinate system to reflect the selection of the navigation route of the AUV better. The smaller the angle is, the smaller the included angle between the navigation direction and the target position is. The curve demonstrates that the angle of AUV under the guidance of PPO changes to 0° when the number of steps reaches approximately 480. At this time, the navigation direction is optimal, and there is no obstacle in front of the route; however, the curve of angle has a large lifting movement; hence, AUV does not sail straight as we expected. In comparison with the other two algorithms, the heading angle of AUV more sharply changes, and the navigation path is longer. The angle between the heading of the AUV and the target position under the guidance of SAC and SAC-GAIL is gradually reduced and is finally maintained within 10°. Therefore, the planned path is shorter, which conforms to the rules of reward value setting.

In summary, the decision-making process of AUV directly reflects the demand of the reward function. The reason for the short backward movement is to complete the turning action at low velocity, reduce the angle between the heading and the target position, and increase the discount reward value. However, this type of motion cannot be performed for a long time because we give a penalty to the backward movement, and the time and distance are also required to be as short as possible. Therefore, the system outputs the boundary action as much as possible to obtain a larger acceleration and obtain a greater sailing velocity and angular velocity in yaw. Under the constraints of such motion planning, AUV seeks for a path that maximizes the accumulative discount reward value, that is, a complete action decision sequence. In terms of planning results, SAC introduces maximum entropy; thus, it increases the exploration ability of AUV. In the motion planning task, multiple routes can be utilized to avoid obstacles and reach the target point, and the best route and control instruction among them should be chosen. When facing the same task, especially in the case of more constraints, SAC can give full play to its advantages; therefore, it is better than other algorithms. GAIL can also speed up the AUV training speed and reduce the training time without affecting the planning effect of the SAC algorithm.

## 5. Conclusions

This research proposes an end-to-end AUV motion planning method based on the SAC algorithm to solve the problems of poor exploration ability, single strategy, and high training cost in AUV motion planning task and overcome certain difficulties, such as multiple constraints and sparse reward environment. The system directly maps the state information of the AUV and the environment into the control instructions of the AUV, which realizes the end-to-end processing of the information. The method of GAIL is also used to assist its training, and the AUV is guided in the form of experts, reducing the cost of interaction between the AUV and the environment and solving the difficult and time-consuming problem of learning strategies from scratch in reinforcement learning. We also design a comprehensive reward function related to various factors, such as velocity, distance, and heading angle, to encourage the AUV to smoothly reach the target position and make the distance and time as optimal as possible. Finally, the motion planning effects of the PPO, SAC, and SAC-GAIL algorithms are compared on the basis of the Unity simulation platform. The results show that the end-to-end motion planning system proposed in this research has better decision-making ability in the process of navigation, shorter route, less time consumption, and smoother trajectory. After the introduction of GAIL, the convergence speed of training has been greatly improved, although the effect of AUV motion planning has not been greatly enhanced.

The motion planning method proposed in this work still has some shortcomings. GAIL improves the convergence speed of SAC, but does not significantly enhance the motion planning effect of SAC. Even when the target point randomly appears in some positions, the planning time and distance are not as good as the SAC algorithm. First, the training under the same number of training steps and hardware conditions takes a substantial amount of time to complete because the neural network structure becomes more complex. Second, expert samples are difficult to obtain in many tasks. On the basis of introducing a survivor bias, how to shorten the voyage distance and voyage time at the same time and to coordinate the proportion of GAIL signal and external rewards is a problem to be addressed in the future. 

## Figures and Tables

**Table 1:** The pros and cons of several algorithms.

Algorithm		Pros	Cons
Geometric model search		Simple and easy to implement	Low flexibility, poor real-time performance, lack of intelligent understanding
Rapidly exploring random tree		Search in high-dimensional space quickly and efficiently	The planning result is not optimal
Artificial potential field method		Simple operation and high real-time performance	Local optimal problem
Curve interpolation		The algorithm is intuitive, and the planned trajectory is also very flat	A large amount of calculation and low real-time performance
Genetic algorithm		Good robustness and easy to combine with other methods	Slow convergence speed
RL	DQN	Solve the problem of dimension disaster when the state and action space is too large	Single-step update, only applicable to discrete spaces and actions
	DDPG	Solve the motion problem in the continuous action space	The randomness of the strategy is low, and the convergence effect is poor
	PPO	It can face both discrete control and continuous control	On policy, low sample efficiency
	SAC	Off policy, less sensitive to different hyperparameter values, strong exploration ability and high robustness	Learning a policy from scratch is difficult and time-consuming
Imitation learning		Fast convergence speed with expert guidance	Good expert samples are difficult to obtain

**Table 2:** AUV parameters.

Parameter	Value
m	45 kg
L	1.46 m
$X_{\dot{u}}$ ^1^	−1.5777 × 10^−3^
$Y_{\dot{v}}$	−3.0753 × 10^−2^
$Y_{\dot{r}}$	9.4196 × 10^−4^
$N_{\dot{r}}$	−1.012 × 10^−1^
$X_{u\left|u\right|}$	−5.9 × 10^−3^
$Y_{v\left|v\right|}$	−1.6687 × 10^−1^
$Y_{v\left|r\right|}$	0
$Y_{r\left|v\right|}$	0
$Y_{r\left|r\right|}$	1.258 × 10^−1^
$N_{v\left|v\right|}$	0
$N_{v\left|r\right|}$	0
$N_{r\left|v\right|}$	0
$N_{r\left|r\right|}$	−1.2432 × 10^−1^

^1^$X_{*},\;Y_{*},\;N_{*}$ denotes the hydrodynamic coefficient.

**Table 3:** Sensor parameters.

Sensors	Parameter	Value
DVL	Frequency	600 kHz
	Accuracy	1% ± 1 mm/s
	Maximum Velocity	±20 knots
OAS	Sharp Angle	$17^{\circ }\pm 2^{\circ }$
	Range	0.6~120 m
	Reliable Range	1~20 m

**Table 4:** Effect of stack.

Steps	Observation Vector	Observation Vector After Stacking ^1^
Step 1	[0.6]	[0.6, 0.0, 0.0]
Step 2	[0.4]	[0.6, 0.4, 0.0]
Step 3	[0.9]	[0.9, 0.4, 0.6]
Step 4	[0.2]	[0.2, 0.9, 0.4]

^1^ We set the size of the stack to three here.

**Table 5:** Position and collision size of each element in the environment.

Element	Position	Collision Size	
AUV	(0,0)	Same size as actual model	
Goal	$\left(x_{goal},k\sqrt{35^{2}-x_{goal}^{2}}\right)$ $x_{goal}\in random\left(-1,1\right)*35\;k=random(+1/-1)$	Radius = 1	
/		Length	Width
Cruciform Obstacle 1	(16.5, 16.5)	9	0.6
Cruciform Obstacle 2	(16.5, −16.5)	9	0.6
Cruciform Obstacle 3	(−16.5, −16.5)	9	0.6
Cruciform Obstacle 4	(−16.5, 16.5)	9	0.6
Rectangular Obstacle 1	(15, 45)	8	1
Rectangular Obstacle 2	(45, 15)	8	1
Rectangular Obstacle 3	(45, −15)	8	1
Rectangular Obstacle 4	(15, −45)	8	1
Rectangular Obstacle 5	(−15, −45)	8	1
Rectangular Obstacle 6	(−45, −15)	8	1
Rectangular Obstacle 7	(−45, 15)	8	1
Rectangular Obstacle 8	(−15, 45)	8	1

**Table 6:** Parameters of the reward value.

Reward Parameter	Value
$r_{1}$	+20
$r_{2}$	−20
$k_{1}$	10 × 10^−1^
$k_{2}$	10 × 10^−2^
$k_{3}$	π/(180 × 25,000)
$r_{3}$	−10 × 10^−5^

**Table 7:** Specific experimental parameters.

Parameters	PPO	SAC
batch_size (number of experiences in each iteration of gradient descent)	2048	256
buffer_size (number of experiences to collect before updating the policy model)	20,480	1,000,000
learning rate	0.0003	0.0003
learning_rate_schedule (determines how the learning rate changes over time)	Linear decay	Fixed constant
β (strength of the entropy regularization in PPO)	0.001	/
ε (influences how rapidly the policy can evolve during training in PPO)	0.2	/
λ (regularization parameter used when calculating the Generalized Advantage Estimate in PPO)	0.95	/
num_epoch (number of passes to make through the experience buffer when performing gradient descent optimization in PPO)	3	/
tau (how aggressively to update the target network used for bootstrapping value estimation in SAC)	/	0.005
steps_per_update (average ratio of actions taken to updates made of the agent’s policy in SAC)	/	10.0
reward_signal_steps_per_update (number of steps per mini batch sampled and used for updating the reward signals in SAC)	/	10.0
hidden_units (number of units in the hidden layers of the neural network)	256	256
num_layers (the number of hidden layers in the neural network)	2	2
γ (discount factor for future rewards)	0.99	0.99
max_steps/per episode	16,000	16,000
max_steps	8,000,000	8,000,000

**Table 8:** Distance and time information of the trajectory planned.

Target Coordinates	Index	PPO	SAC	SAC-GAIL
(−15.4, 31.4)	Distance (m)	68.48	39.10	38.01
	Time (s)	42.34	25.50	26.42
(16.8, 30.7)	Distance (m)	49.27	35.65	35.55
	Time (s)	27.90	20.52	20.32
(25.0, 24.5)	Distance (m)	51.30	40.30	40.76
	Time (s)	28.72	21.72	22.08
(−29.2, −19.3)	Distance (m)	75.10	39.96	37.60
	Time (s)	50.16	31.56	32.40
(−11.6, −33.0)	Distance (m)	46.04	38.71	38.20
	Time (s)	26.04	23.76	23.78
(23.1, −26.3)	Distance (m)	64.00	41.03	40.92
	Time (s)	43.72	22.56	22.04

## Data Availability

A simple video demonstration of the system’s work can be found at the link: https://youtu.be/E7ncKYGgNCc (accessed on 25 August 2021).

## Appendix A

**Proof of Theorem** **A1.** We can decompose the expected return $\max E_{\rho_{\pi }}\left[\sum_{t=0}^{T}r\left(s_{t},a_{t}\right)\right]$ using the idea of Dynamic Programming. We can maximize the return at different steps backward in time because the policy $\pi_{t}$ at time *t* has no effect on the policy at the earlier time step $\pi_{t-1}$

(A1) $$\underset{last}{\underset{︸}{\underset{\pi_{0}}{\max}\left(E\left[r\left(s_{0},a_{0}\right)\right]+\underset{second\;last}{\underset{︸}{\underset{\pi_{1}}{\max}\left(E\left[\ldots \right]\right)+\underset{first}{\underset{︸}{\underset{\pi_{T}}{\max}E\left[r\left(s_{T},a_{T}\right)\right]}}}}\right)}},$$

where we assume that γ=1 for the convenience of calculation. Now, we start the optimization from the last timestep *T*:

(A2) $$\max E_{\left(s_{T},a_{T}\right)∼\rho_{\pi }}\left[r\left(s_{T},a_{T}\right)\right]\;s.t.\forall t,H\left(\pi_{T}\right)\geq H_{0}.$$

We can choose the Lagrangian multiplier method to solve extreme value problems with constraints. Now, we need to construct a Lagrangian expression:

(A3) $$L\left(\pi_{T},\alpha_{T}\right)=E_{\rho_{\pi }}\left[\sum_{t=0}^{T}r\left(s_{t},a_{t}\right)\right]+\alpha_{T}\left[H\left(\pi_{T}\right)-H_{0}\right],$$

where $\alpha_{T}$ is the Lagrangian multiplier. We can limit α≥0 to maximize $L\left(\pi_{T},\alpha_{T}\right)$. If the constraint is satisfied (i.e., $H\left(\pi_{t}\right)\geq H_{0}$), then we can set $\alpha_{T}=0$ because we have no control over the value of $H\left(\pi_{T}\right)-H_{0}$. Therefore, we can convert the problem of (A2) into a dual problem:

(A4) $$\begin{array}{c} \max E_{\left(s_{T},a_{T}\right)∼\rho_{\pi }}\left[r\left(s_{T},a_{T}\right)\right]\;s.t.\forall t,H\left(\pi_{T}\right)\geq H_{0}\\ ⇓\\ \underset{\alpha_{T}\geq 0}{\min}\underset{\pi_{T}}{\max}L\left(\pi_{T},\alpha_{T}\right) \end{array}.$$

Furthermore, we replace $L\left(\pi_{T},\alpha_{T}\right)$ with Equation (A3):

(A5) $$\underset{\pi_{T}}{\max}E_{\left(s_{T},a_{T}\right)∼\rho_{\pi }}\left[r\left(s_{T},a_{T}\right)\right]=\underset{\alpha_{T}\geq 0}{\min}\underset{\pi_{T}}{\max}E_{\left(s_{T},a_{T}\right)∼\rho_{\pi }}\left[r\left(s_{T},a_{T}\right)+\alpha_{T}H\left(\pi_{T}\right)\right]-\alpha_{T}H_{0}.$$

In Equation (A5), given a fixed value of $\alpha_{T}$, we can get the optimal policy $\pi_{T}^{*}$ to maximize $L\left(\pi_{T}^{*},\alpha_{T}\right)$. Then, we plug in $\pi_{T}^{*}$ and compute $\alpha_{T}^{*}$ that minimizes $L\left(\pi_{T}^{*},\alpha_{T}\right)$. Hence,

(A6) $$\pi_{T}^{*}=\underset{\pi_{T}}{\arg\max}E_{\left(s_{T},a_{T}\right)∼\rho_{\pi }}\left[r\left(s_{T},a_{T}\right)+\alpha_{T}H\left(\pi_{T}\right)-\alpha_{T}H_{0}\right],$$

(A7) $$\alpha_{T}^{*}=\underset{\alpha_{T}\geq 0}{\arg\min}E_{\left(s_{T},a_{T}\right)∼\rho_{\pi^{*}}}\left[\alpha H\left(\pi_{T}^{*}\right)-\alpha_{T}H_{0}\right].$$

Then, according to soft *Q* value Equation (21):

(A8) $$Q_{T-1}\left(s_{T-1},a_{T-1}\right)=r\left(s_{T-1},a_{T-1}\right)+E_{\rho_{\pi }}\left[Q\left(s_{T},a_{T}\right)-\alpha_{T}\log\pi_{t}\left(a_{T}|s_{T}\right)\right].$$

Variable *T* is the last moment; hence, $Q\left(s_{T},a_{T}\right)=r\left(s_{T},a_{T}\right)$. The previous expression will be transformed to:

(A9) $$Q_{T-1}\left(s_{T-1},a_{T-1}\right)=r\left(s_{T-1},a_{T-1}\right)+E_{\rho_{\pi }}\left[r\left(s_{T},a_{T}\right)+\alpha_{T}H\left(\pi_{T}\right)\right],$$

(A10) $$Q_{T-1}^{*}\left(s_{T-1},a_{T-1}\right)=r\left(s_{T-1},a_{T-1}\right)+\underset{\pi_{T}}{\max}\left[r\left(s_{T},a_{T}\right)+\alpha_{T}^{*}H\left(\pi_{T}^{*}\right)\right].$$

We take one step further back to the time step *T*−1, γ=1, according to Equation (A10):

(A11) $$\begin{array}{l} \underset{\pi_{T-1}}{\max}\left(E\left[r\left(s_{T-1},a_{T-1}\right)\right]+\underset{\pi_{T}}{\max}r\left(s_{T},a_{T}\right)\right)\\ =\underset{\pi_{T-1}}{\max}\left[Q_{T-1}^{*}\left(s_{T-1},a_{T-1}\right)-\alpha_{T}^{*}H\left(\pi_{T}^{*}\right)\right]\;s.t.\;H\left(\pi_{T-1}\right)-H_{0}\geq 0\\ =\underset{\alpha_{T-1}\geq 0}{\min}\underset{\pi_{T-1}}{\max}\left[Q_{T-1}^{*}\left(s_{T-1},a_{T-1}\right)+\alpha_{T-1}H\left(\pi_{T-1}\right)-\alpha_{T-1}H_{0}\right]-\alpha_{T}^{*}H\left(\pi_{T}^{*}\right) \end{array}.$$

At this time, we can get the optimal policy $\pi_{T-1}^{*}$ and the optimal dual parameter $\alpha_{T-1}^{*}$ at *T*−1 time:

(A12) $$\pi_{T-1}^{*}=\underset{\pi_{T-1}}{\arg\max}E_{\left(s_{T-1},a_{T-1}\right)∼\rho_{\pi }}\left[Q_{T-1}^{*}\left(s_{T-1},a_{T-1}\right)+\alpha_{T-1}H\left(\pi_{T-1}\right)-\alpha_{T-1}H_{0}\right],$$

(A13) $$\alpha_{T-1}^{*}=\underset{\alpha_{T-1}\geq 0}{\arg\min}E_{\left(s_{T-1},a_{T-1}\right)∼\rho_{\pi^{*}}}\left[\alpha_{T-1}H\left(\pi_{T-1}^{*}\right)-\alpha_{T-1}H_{0}\right].$$

Finally, we can obtain the objective function of temperature parameter α by repeating formulas (A12) and (A13):

(A14) $$J\left(\alpha \right)=E_{a_{t}∼\pi_{t}}\left[-\alpha \log\pi_{t}\left(a_{t}|s_{t}\right)-\alpha H_{0}\right].$$

 □

## References

[1] Mnih V., Kavukcuoglu K., Silver D., Graves A., Antonoglou I., Wierstra D., Riedmiller M. (2013). Playing atari with deep reinforcement learning. arXiv.

[2] Dijkstra E.W. (1959). A note on two problems in connexion with graphs. Numer. Math..

[3] Scharff Willners J., Gonzalez-Adell D., Hernández J.D., Pairet È., Petillot Y. (2021). Online 3-Dimensional Path Planning with Kinematic Constraints in Unknown Environments Using Hybrid A* with Tree Pruning. Sensors.

[4] Cui R., Li Y., Yan W. (2015). Mutual information-based multi-AUV path planning for scalar field sampling using multidimensional RRT. IEEE Trans. Syst. Man Cybern. Syst..

[5] Fan X., Guo Y., Liu H., Wei B., Lyu W. (2020). Improved artificial potential field method applied for AUV path planning. Math. Probl. Eng..

[6] Zeng Z., Sammut K., He F., Lammas A. Efficient path evaluation for AUVs using adaptive B-spline approximation. Proceedings of the IEEE Oceans.

[7] Cai W., Zhang M., Zheng Y.R. (2017). Task assignment and path planning for multiple autonomous underwater vehicles using 3D dubins curves. Sensors.

[8] Sutton R.S., Barto A.G. (2018). Reinforcement Learning: An Introduction.

[9] Wang L., Kan J., Guo J., Wang C. (2019). 3D path planning for the ground robot with improved ant colony optimization. Sensors.

[10] Hao K., Zhao J., Yu K., Li C., Wang C. (2020). Path planning of mobile robots based on a multi-population migration genetic algorithm. Sensors.

[11] Bai X., Yan W., Ge S.S., Cao M. (2018). An integrated multi-population genetic algorithm for multi-vehicle task assignment in a drift field. Inf. Sci..

[12] Bai X., Yan W., Cao M. (2017). Clustering-based algorithms for multivehicle task assignment in a time-invariant drift field. IEEE Robot. Autom. Lett..

[13] Li J., Wang H. Research on AUV Path Planning Based on Improved Ant Colony Algorithm. Proceedings of the 2020 IEEE International Conference on Mechatronics and Automation (ICMA).

[14] Camci E., Kayacan E. (2019). End-to-End Motion Planning of Quadrotors Using Deep Reinforcement Learning. arXiv.

[15] Doukhi O., Lee D. (2021). Deep Reinforcement Learning for End-to-End Local Motion Planning of Autonomous Aerial Robots in Unknown Outdoor Environments: Real-Time Flight Experiments. Sensors.

[16] Cheng Y., Zhang W. (2018). Concise deep reinforcement learning obstacle avoidance for underactuated unmanned marine vessels. Neurocomputing.

[17] Sun Y., Ran X., Zhang G., Xu H., Wang X. (2020). AUV 3D path planning based on the improved hierarchical deep Q network. J. Mar. Sci. Eng..

[18] Sun Y., Cheng J., Zhang G., Xu H. (2019). Mapless motion planning system for an autonomous underwater vehicle using policy gradient-based deep reinforcement learning. J. Intell. Robot. Syst..

[19] Butyrev L.T., Mutschler C. (2019). Deep reinforcement learning for motion planning of mobile robots. arXiv.

[20] Haarnoja T., Zhou A., Abbeel P., Levine S. Soft actor-critic: Off-policy maximum entropy deep reinforcement learning with a stochastic actor. Proceedings of the PMLR.

[21] Haarnoja T., Zhou A., Hartikainen K., Tucker G., Ha S., Tan J., Kumar V., Zhu H., Gupta A., Abbeel P. (2018). Soft actor-critic algorithms and applications. arXiv.

[22] Prianto E., Kim M., Park J., Bae J., Kim J. (2020). Path Planning for Multi-Arm Manipulators Using Deep Reinforcement Learning: Soft Actor–Critic with Hindsight Experience Replay. Sensors.

[23] Wong C., Chien S., Feng H., Aoyama H. (2021). Motion Planning for Dual-Arm Robot Based on Soft Actor-Critic. IEEE Access.

[24] Liu Q., Li Y., Liu L. A 3D Simulation Environment and Navigation Approach for Robot Navigation via Deep Reinforcement Learning in Dense Pedestrian Environment. Proceedings of the 2020 IEEE 16th International Conference on Automation Science and Engineering (CASE).

[25] Cheng Y., Song Y. Autonomous Decision-Making Generation of UAV based on Soft Actor-Critic Algorithm. Proceedings of the 2020 39th Chinese Control Conference (CCC).

[26] Gupta A., Khwaja A.S., Anpalagan A., Guan L., Venkatesh B. (2020). Policy-Gradient and Actor-Critic Based State Representation Learning for Safe Driving of Autonomous Vehicles. Sensors.

[27] Chen J., Li S.E., Tomizuka M. Interpretable end-to-end urban autonomous driving with latent deep reinforcement learning. Proceedings of the IEEE Transactions on Intelligent Transportation Systems.

[28] Ahmad T., Ashraf A., Truscan D., Domi A., Porres I. (2020). Using deep reinforcement learning for exploratory performance testing of software systems with multi-dimensional input spaces. IEEE Access.

[29] Baram N., Anschel O., Caspi I., Mannor S. End-to-end differentiable adversarial imitation learning. Proceedings of the PMLR.

[30] Pomerleau D.A. (1991). Efficient training of artificial neural networks for autonomous navigation. Neural Comput..

[31] Giusti A., Guzzi J., Cireşan D.C., He F.-L., Rodríguez J.P., Fontana F., Faessler M., Forster C., Schmidhuber J., Di Caro G. (2015). A machine learning approach to visual perception of forest trails for mobile robots. IEEE Robot. Autom. Lett..

[32] Bojarski M., Del Testa D., Dworakowski D., Firner B., Flepp B., Goyal P., Jackel L.D., Monfort M., Muller U., Zhang J. (2016). End to end learning for self-driving cars. arXiv.

[33] Ross S.E.P., Bagnell D. Efficient reductions for imitation learning. Proceedings of the JMLR Workshop and Conference Proceedings.

[34] Ross S.E.P., Gordon G., Bagnell D. A reduction of imitation learning and structured prediction to no-regret online learning. Proceedings of the JMLR Workshop and Conference Proceedings.

[35] Shimodaira H. (2000). Improving predictive inference under covariate shift by weighting the log-likelihood function. J. Stat. Plan. Inference.

[36] Ng A.Y., Russell S.J. Algorithms for inverse reinforcement learning. Proceedings of the ICML.

[37] Ziebart B.D., Maas A.L., Bagnell J.A., Dey A.K. Maximum entropy inverse reinforcement learning. Proceedings of the AAAI.

[38] Ratliff N.D., Silver D., Bagnell J.A. (2009). Learning to search: Functional gradient techniques for imitation learning. Auton. Robot..

[39] Goodfellow I., Pouget-Abadie J., Mirza M., Xu B., Warde-Farley D., Ozair S., Courville A., Bengio Y. (2014). Generative adversarial nets. Adv. Neural Inf. Process. Syst..

[40] Brock A., Donahue J., Simonyan K. (2018). Large scale GAN training for high fidelity natural image synthesis. arXiv.

[41] Ho J., Ermon S. (2016). Generative adversarial imitation learning. Adv. Neural Inf. Process. Syst..

[42] Ho J., Gupta J., Ermon S. Model-free imitation learning with policy optimization. Proceedings of the PMLR.

[43] Merel J., Tassa Y., TB D., Srinivasan S., Lemmon J., Wang Z., Wayne G., Heess N. (2017). Learning human behaviors from motion capture by adversarial imitation. arXiv.

[44] Peng X.B., Kanazawa A., Toyer S., Abbeel P., Levine S. (2018). Variational discriminator bottleneck: Improving imitation learning, inverse rl, and gans by constraining information flow. arXiv.

[45] Karimshoushtari M., Novara C., Tango F. (2021). How Imitation Learning and Human Factors Can Be Combined in a Model Predictive Control Algorithm for Adaptive Motion Planning and Control. Sensors.

[46] Zhou Y., Fu R., Wang C., Zhang R. (2020). Modeling Car-Following Behaviors and Driving Styles with Generative Adversarial Imitation Learning. Sensors.

[47] Fossen T.I. (2011). Handbook of Marine Craft Hydrodynamics and Motion Control.

[48] Kober J., Bagnell J.A., Peters J. (2013). Reinforcement learning in robotics: A survey. Int. J. Robot. Res..

[49] Chaffre T., Moras J., Chan-Hon-Tong A., Marzat J. (2020). Sim-to-real transfer with incremental environment complexity for reinforcement learning of depth-based robot navigation. arXiv.

[50] Bellman R. (1966). Dynamic programming. Science.

[51] Haarnoja T., Tang H., Abbeel P., Levine S. Reinforcement learning with deep energy-based policies. Proceedings of the PMLR.

[52] Bhattacharyya R., Wulfe B., Phillips D., Kuefler A., Morton J., Senanayake R., Kochenderfer M. (2020). Modeling human driving behavior through generative adversarial imitation learning. arXiv.

[53] Torabi F., Warnell G., Stone P. (2018). Generative adversarial imitation from observation. arXiv.

[54] Littman M.L. (2015). Reinforcement learning improves behaviour from evaluative feedback. Nature.

